# Metabolic flexibility allows bacterial habitat generalists to become dominant in a frequently disturbed ecosystem

**DOI:** 10.1038/s41396-021-00988-w

**Published:** 2021-05-03

**Authors:** Ya-Jou Chen, Pok Man Leung, Jennifer L. Wood, Sean K. Bay, Philip Hugenholtz, Adam J. Kessler, Guy Shelley, David W. Waite, Ashley E. Franks, Perran L. M. Cook, Chris Greening

**Affiliations:** 1 Department of Microbiology, Biomedicine Discovery Institute, Clayton, VIC Australia; 2 School of Biological Sciences, Monash University, Clayton, VIC Australia; 3 Department of Natural Resources Sciences, McGill University, Sainte-Anne-de-Bellevue, QC Canada; 4 Department of Physiology, Anatomy and Microbiology, La Trobe University, Bundoora, VIC Australia; 5 Australian Centre for Ecogenomics, School of Chemistry and Molecular Biosciences, The University of Queensland, St Lucia, QLD Australia; 6 Water Studies Centre, School of Chemistry, Monash University, Clayton, VIC Australia; 7 School of Earth, Atmosphere and Environment, Monash University, Clayton, VIC Australia; 8 School of Biological Sciences, University of Auckland, Auckland, New Zealand

**Keywords:** Microbial ecology, Microbial communities

## Abstract

Ecological theory suggests that habitat disturbance differentially influences distributions of habitat generalist and specialist species. While well-established for macroorganisms, this theory has rarely been explored for microorganisms. Here we tested these principles in permeable (sandy) sediments, ecosystems with much spatiotemporal variation in resource availability and physicochemical conditions. Microbial community composition and function were profiled in intertidal and subtidal sediments using 16S rRNA gene amplicon sequencing and metagenomics, yielding 135 metagenome-assembled genomes. Community composition and metabolic traits modestly varied with sediment depth and sampling date. Several taxa were highly abundant and prevalent in all samples, including within the orders Woeseiales and Flavobacteriales, and classified as habitat generalists; genome reconstructions indicate these taxa are highly metabolically flexible facultative anaerobes and adapt to resource variability by using different electron donors and acceptors. In contrast, obligately anaerobic taxa such as sulfate reducers and candidate lineage MBNT15 were less abundant overall and only thrived in more stable deeper sediments. We substantiated these findings by measuring three metabolic processes in these sediments; whereas the habitat generalist-associated processes of sulfide oxidation and fermentation occurred rapidly at all depths, the specialist-associated process of sulfate reduction was restricted to deeper sediments. A manipulative experiment also confirmed habitat generalists outcompete specialist taxa during simulated habitat disturbance. Together, these findings show metabolically flexible habitat generalists become dominant in highly dynamic environments, whereas metabolically constrained specialists are restricted to narrower niches. Thus, an ecological theory describing distribution patterns for macroorganisms likely extends to microorganisms. Such findings have broad ecological and biogeochemical ramifications.

## Introduction

In macroecology, species are broadly classified as habitat generalists and specialists depending on their niche breadth [1, 2]. Both deterministic and stochastic factors control the differential distributions of such species and in turn the maintenance of diversity [3, 4]. With respect to deterministic factors, a pervasive ecological theory is that generalists and specialists differ in performance traits, for example resource utilization. Habitat generalists are thought to be more versatile but less efficient than habitat specialists, whereas specialists perform fewer activities more effectively; by extension, it can be predicted that specialists will outcompete generalists in their optimal habitats, whereas generalists will be favored in environments with high spatial and temporal heterogeneity [1, 5]. The degree of habitat specialization may also predict responses to disturbance, given increasing evidence that both natural and anthropogenic habitat disturbance favors generalists and promotes homogenization of community composition [6–8]. Other factors, notably dispersal traits and life history strategies, also influence distribution patterns [3, 9]. While these tenets are well-established for animals and plants, few studies have extended them to microbial communities [10–12].

The key ecological processes governing macroorganism community assembly are thought to extend to microorganisms. Both environmental selection and stochastic factors such as dispersal limitation contribute to microbial community assembly [13–15]. These processes lead to an uneven prevalence of microbial taxa across ecosystems, with most community members having low to intermediate ranges (habitat specialists) and a small proportion of taxa tending to be highly prevalent and often abundant across space and time (habitat generalists) [16–18]. The performance traits that differentiate microbial habitat generalists and specialists, including those that allow key taxa to dominate across a wide variety of habitats, have been scarcely explored. It is probable that, like macroorganisms, a key factor that governs distribution patterns is the capacity and efficiency of resource utilization. In this regard, an important trait that distinguishes microorganisms is metabolic versatility [19]; whereas plants and animals are respectively restricted to photoautotrophic and chemoheterotrophic growth, many microorganisms can use multiple energy sources, carbon sources, and electron acceptors either simultaneously or alternately [12]. Likewise, the capacity for microorganisms to transition between active and dormant states contributes to the maintenance of diversity [20, 21]. It is increasingly realized that such flexibility in resource usage contributes to the dominance of certain taxa, but it remains unclear whether metabolic flexibility is a general feature of microbial habitat generalists [22–29].

Permeable (sandy) sediments are ideal sites to explore the concepts of habitat generalism and specialism in microorganisms. These ecosystems, spanning at least half the continental shelf, are important regulators of oceanic biogeochemical cycling and primary production [30–32]. Their uppermost sediments (i.e., mixing layer) are continuously disrupted, primarily due to porewater advection in conjunction with tide- and wave-driven hydrodynamic disturbance [33–35]. As a result, microorganisms living in mixing layers experience large variations in the availability of light, oxygen, and other resources across short spatial and temporal scales [30, 36]. In contrast, microbial communities in the deeper sediment layers are infrequently disturbed and are generally exposed to dark anoxic conditions [37]. Overall, permeable sediments are known to harbor abundant, diverse, and active microbial communities [27, 38–42]. Previous studies have indicated that there is a rapid community turnover across depth and season in Wadden Sea sediments [38]. However, some lineages such as the Woeseiaceae appear to be abundant and prevalent residents of all permeable sediments sampled worldwide [23, 43, 44]. The functional basis for their dominance is unclear. We have recently published evidence that metabolic flexibility, including the ability of bacteria to shift from aerobic respiration to hydrogenogenic fermentation in response to oxic-anoxic transitions, is an important factor controlling the ecology and biogeochemistry of the communities in the mixing layer [43, 45]. Due to these dynamics, fermentation and respiratory processes are uncoupled in well-mixed permeable sediments, in contrast to most sedimentary ecosystems where these processes are closely coupled and follow a redox cascade [43, 45, 46].

In this study, we investigated the spatiotemporal distributions and metabolic traits of habitat generalists and specialists in permeable sediments from Middle Park Beach, Port Philip Bay, Australia. Given the above considerations, we hypothesized that the mixing and deep layers of permeable sediments would select for different metabolic traits. The mixing layer, reflecting its spatiotemporal variability, would select for habitat generalists with broad metabolic capabilities. In contrast, the less frequently disturbed deep layer would allow establishment of relative habitat specialists with constrained but efficient anaerobic lifestyles. To test this, we used high-resolution community profiling to determine the spatiotemporal distribution of bacterial and archaeal communities in shallow, intermediate, and deep sands. While various concepts and definitions have been developed to describe ecological specialization [47, 48], here we defined species as ‘habitat generalists’ and ‘habitat specialists’ based on their distribution (i.e., coefficient of variance of their relative abundance as described [6]), rather than by functional traits. We used genome-resolved metagenomics, biogeochemical assays, and phylogenetic analysis to determine the metabolic capabilities of the most dominant habitat generalists and specialists, revealing most habitat generalists were highly metabolically flexible. Perturbation experiments were used to validate the environment-competition dynamics predicted for habitat generalists and specialists from our hypothesis.

## Materials and methods

### Sampling of permeable sediments

Permeable sediments were sampled from Middle Park Beach, Port Phillip Bay. Samples for microbial community profiling were collected from the same location (37.851342°S, 144.954377°E) over eight different dates over the course of a year (A: 28/10/2016; B: 13/12/2016; C: 19/1/2017; D: 28/3/2017; E: 9/5/2017; F: 30/6/2017; G: 23/8/2017; H: 19/10/2017). Cores of 30 cm were used to collect sediments from the subtidal zone (~1 m deep at low tide) and intertidal zone (~1 m deep at high tide); one sediment core was collected at high tide and low tide respectively on the same sampling date. The sampled sediments mainly comprise sands and gravels with a median grain size of ~0.55 mm [49]. Tide and weather details for each of the sampling dates are provided in Table S1. Cores were kept on ice until delivery to the laboratory and were then immediately sectioned into shallow (0–3 cm), intermediate (14–17 cm), and deep (27–30 cm) samples. All samples were subsequently stored at −20 °C until further processing.

### Amplicon sequencing

For amplicon sequencing, total community DNA was extracted from 0.25 g of sediment using the modified Griffith’s protocol [50]. The yield, purity, and integrity of DNA from each extraction was confirmed using a Qubit Fluorometer, Nanodrop 1000 Spectrophotometer, and agarose gel electrophoresis, respectively. For each sample, the V4 hypervariable region for 16S rRNA gene was amplified using the universal Earth Microbiome Project primer pairs F515 and R806 [51] and subjected to Illumina paired-end sequencing at the Australian Centre for Ecogenomics, University of Queensland. Paired-end raw reads were demultiplexed and adapter sequences were trimmed, yielding 1,362,535 reads across all samples. Forward and reverse sequences were merged using the q2-vsearch plugin [52]. A quality filtering step was applied using a sliding window of four bases with an average base call accuracy of 99% (Phred score 20). The reads were truncated down to 250 base pairs to remove low quality reads before de-noising using the deblur pipeline [53] in QIIME 2 [54]. Six samples with read counts <1000 were removed for downstream analysis, leaving a total of 42 samples. 270 ASVs that only occurred once (i.e., singletons) and 301 ASVs flagged using the decontam R package [55] were removed from the dataset, resulting in the retention of a total of 12,265 ASVs (Table S2). For taxonomic assignment, all reference reads that matched the F515/R806 primer pair were extracted from the Genome Taxonomy Database (GTDB) release 89 [56] and used to train a naïve bayes classifier by using the fit-classifier-naive-bayes function with default parameters (Table S3).

### Biodiversity analysis

All statistical analysis and visualizations were performed with R software version 4.0.2 (June 2020) using the packages phyloseq [57], vegan [58], and ggplot2 [59]. Prior to statistical analysis, all sequences were rarefied at 5000 sequences per sample. Alpha diversity was calculated using several metrics, including Shannon index, which measures both species richness and evenness. We tested for significant differences in Shannon index between depth, tidal zone, and date using a one-way ANOVA with Tukey’s *post hoc* tests (*p* < 0.05). Beta diversity was calculated using weighted UniFrac distances [60] of log_10_-transformed data and visualized using principal coordinate analysis (PCoA) and nonmetric dimensional scaling (NMDS). A pairwise analysis of similarities (ANOSIM) was used to test for significant differences in community similarity between depths, tidal zone, and date. First, permutational multivariate analysis of variance was performed using 999 permutations to test for significant differences. Second, a beta dispersion test (PERMDISP) was used to ascertain if observed differences were influenced by dispersion. The occupancy of each ASV, i.e., number or proportion of samples in which they were present, was computed by the average of 200 different rarefactions of the datasets at 5000 sequences per sample. We also applied the new incidence-based diversity metric zeta diversity, which quantifies the average number of ASVs shared across multiple samples. Zeta decline, which compared the average number of shared taxa between two and eight samples at each depth, was computed using the function *Zeta.decline.mc* in zetadiv [61] with 1000 bootstraps. Similarly, zeta temporal decay was computed using the function *Zeta.ddecay* with 1000 bootstraps to show turnover of communities with sampling time at each depth. Values were normalized by the Jaccard method to account for sample richness differences. Based on AIC values and *p* values, zeta decline better fitted a power law rather than exponential form (Table S4). Thus, a power law regression was applied to visualize both zeta decline and zeta temporal decay.

### Classification of habitat generalists and specialists

The degree of habitat specialization of each taxon (i.e., whether they were relative ‘habitat generalists’ or ‘habitat specialists’) was calculated based on their frequency in the 16S rRNA gene amplicon profiles of the 48 samples from the Middle Park Beach cores (Table S5). Specifically, we calculated a specialization index for all taxonomically assigned orders, families, and genera as previously described [6]. This specialization index is calculated as the coefficient of variation (i.e., standard deviation divided by mean) of taxon densities across samples. It also includes a bias correction procedure to correct for undersampled (rare) taxa whereby, assuming taxa follow a Poisson distribution, the expected bias can be calculated as:

$$\surd \frac{{{\mathrm{number}}\;{\mathrm{of}}\;{\mathrm{habitat}}\;{\mathrm{classes}}\;({\mathrm{K}})}}{{{\mathrm{total}}\;{\mathrm{individuals}}\;{\mathrm{in}}\;{\mathrm{a}}\;{\mathrm{given}}\;{\mathrm{taxa}}\;({\mathrm{N}})}}$$

Thus, bias values will decrease with increasing sampling efforts (N). Final SI scores are calculated as raw SI score minus the expected SI bias. While habitat specialization occurs on a spectrum, we took the mean community specialization index (order level: 0.64; family level: 0.65, genus level: 0.66) as a cut-off below which to qualitatively define taxa as relative ‘habitat generalists’. It should be noted that the data from metagenomic sequencing and ex situ manipulative experiment were not used to classify habitat specialization; instead, genome-resolved metagenomics was used to infer metabolic traits of the relative ‘habitat generalists’ and ‘habitat specialists’, whereas ex situ manipulative experiments were used to test whether taxa behaved as predicted following simulated environmental disturbance.

### Quantitative PCR

Quantitative PCR (qPCR) was used to absolutely quantify the copy number of the 16S rRNA genes in the samples. Amplifications were performed using a 96-well plate in a pre-heated LightCycler 480 Instrument II (Roche, Basel, Switzerland). Each well contained a 10 µl reaction mixture comprising 1 µl DNA template, 5 µl Platinum SYBRGreen qPCR SuperMix-UDG with ROX, 0.5 µl each of the universal 16 S rRNA gene V4 primers F515 and R806 (10 µM) [51], and 3 µl UltraPure Water (Thermo Fisher Scientific, Waltham, MA, USA). Each amplification was performed in technical triplicate. Cycling conditions were as follows: 3 min denaturation at 94 °C followed by 40 cycles of 45 s denaturation at 94 °C, 60 s annealing at 50 °C, and 90 s extension at 72 °C. Copy number was quantified against a serially diluted pMA plasmid standard containing a single copy of the *Escherichia coli* 16S rRNA gene. Plasmid dilutions ranged from 10^3^ to 10^8^ copies µl^−1^ and the qPCR amplification efficiency ranged from 85 to 94% (*R*^2^ > 0.99).

### Chlorophyll *a* measurements

Chlorophyll *a* was extracted using a previously described method [62]. Briefly, 5 mL of 90% acetone (v/v) was added to 5 g of sediments in 50 ml Falcon tubes. Samples were then stored overnight in the dark at 4 °C. All samples were subsequently centrifuged at 550 × *g* for 15 min and 3 mL of supernatant was transferred into cuvettes. Chlorophyll absorbance was measured spectrophotometrically using a Hitachi U-2800 spectrophotometer (Hitachi High-Technologies Corporation, Tokyo, Japan) at five different wavelengths (630, 647, 664, 665, and 750 nm). Spectra were read before and after acidification with 10 μL of 1 M HCl (v/v). After calculating the difference in absorbance between the first and second measurement, chlorophyll *a* concentration was determined using the equation of Lorenzen [62].

### Shotgun metagenome sequencing

Table S6 summarizes details of the metagenomic datasets. For this study, we sequenced eight new metagenomes (subtidal deep A, intertidal deep A, subtidal shallow C, intertidal shallow C, subtidal intermediate C, intertidal intermediate C, subtidal deep C, intertidal deep C) and analyzed five previously reported metagenomes (subtidal shallow A, subtidal intermediate A, intertidal shallow A, intertidal intermediate A, flow-through reactor) [43]. DNA was extracted from the 0.3 g of sediment, collected during the October 2016 (A samples) and January 2017 (C samples) field trips, using the MoBio PowerSoil Isolation kit according to manufacturer’s instructions. Metagenomic shotgun libraries were prepared for each sample using the Nextera XT DNA Sample Preparation Kit (Illumina Inc., San Diego, CA, USA) and sequencing was performed on a NextSeq500 platform with a 2 × 150 bp High Output run. Sequencing yielded 574,093,137 read pairs across the eight metagenomes. To supplement the 16S rRNA gene amplicon sequencing data, community profiles in permeable sediments were independently generated from metagenome reads that mapped to the universal single copy ribosomal marker gene *rplP* using SingleM v.0.12.1 (https://github.com/wwood/singlem) (Table S7).

### Shotgun metagenome assembly and binning

The BBDuk function of the BBTools v38.51 (https://sourceforge.net/projects/bbmap/) was used to clip contaminating adapters (k-mer size of 23 and hamming distance of 1), filter PhiX sequences (k-mer size of 31 and hamming distance of 1), and trim bases with a Phred score below 20 from the raw metagenomes. 482,529,838 high-quality read pairs with lengths over 50 bp were retained for downstream analysis. Reads were assembled individually and collectively with MEGAHIT v1.2.9 [63] (--k-min 27, --k-max 127, --k-step 10). Bowtie2 v2.3.5 [64] was used to map short reads back to assembled contigs using default parameters to generate coverage profiles. Subsequently, genomic binning was performed using CONCOCT v1.1.0 [65], MaxBin2 v2.2.6 [66], and MetaBAT2 v2.13 [67] and bins from the same assembly were then dereplicated using DAS_Tool v1.1 [68]. Applying a threshold average nucleotide identity of 99%, bins from different assemblies were consolidated to a non-redundant set of metagenome-assembled genomes (MAGs) using dRep v2.3.2 [69]. Completeness and contamination of MAGs were assessed using CheckM v1.1.2 [70]. In total, 38 high quality (completeness > 90% and contamination < 5%) and 97 medium quality (completeness > 50% and contamination < 10%) [71] MAGs were recovered. Their corresponding taxonomy was assigned by GTDB-Tk v1.0.2 [56] with reference to GTDB r89 [56]. Open reading frames (ORFs) in MAGs were predicted using Prodigal v2.6.3 metagenomic setting [72].

### Shotgun metagenome functional analysis

To estimate the metabolic capability of the sediment communities, metagenomes and derived MAGs were searched against custom protein databases of representative metabolic marker genes (10.26180/c.5230745) using DIAMOND v.0.9.22 [73] (query cover > 80%) with default settings (Table S8–S10). Searches were carried out using all quality-filtered unassembled reads with lengths over 140 bp. In addition, we searched ORFs from the 135 MAGs retrieved from this study and 12 MAGs that were previously reported [43]. These genes are involved in aerobic respiration/detoxification (CoxA, CcoN, CyoA, CydA), oxidative phosphorylation (AtpA), NADH oxidation (NuoF), sulfur cycling (AsrA, FCC, Sqr, DsrA, Sor, SoxB), nitrogen cycling (AmoA, HzsA, NifH, NarG, NapA, NirS, NirK, NrfA, NosZ, NxrA, NorB), iron cycling (Cyc2, OmcB), formate oxidation (FdhA), arsenic cycling (ARO, ArsC), selenium cycling (YgfK), reductive dehalogenation (RdhA), photophosphorylation (PsaA, PsbA, energy-converting microbial rhodopsin), methane cycling (McrA, MmoA, PmoA), hydrogen cycling (large subunit of NiFe-, FeFe-, and Fe-hydrogenases), carbon monoxide oxidation (CoxL, CooS), succinate oxidation (SdhA), fumarate reduction (FrdA), and carbon fixation (AclB, AcsB, HbsC, HbsT, Mcr, RbcL) [74–76]. Results were further filtered based on an identity threshold of 50%, except for group 4 [NiFe]-hydrogenases, [FeFe]-hydrogenases, CoxL, AmoA, NxrA and NuoF (60%), AtpA, YgfK, HbsT, ARO, and PsbA (70%), and PsaA (80%). Subgroup classification of reads was based on the closest match to the sequences in databases. Read counts to each gene were normalized to reads per kilobase per million (RPKM) by dividing the actual read count by the total number of reads (in millions) and then dividing by the individual gene length of the best hit (in kilobases). In order to estimate the gene abundance in the microbial community, high-quality unassembled reads were also screened for the 14 universal single copy ribosomal marker genes used in SingleM v.0.12.1 and PhyloSift [77] by DIAMOND (query cover > 80%, bitscore > 40) and normalized as above. Subsequently, the average gene copy number of a gene in the community can be calculated by dividing the read count for the gene (in RPKM) by the geometric mean of the read count of the 14 universal single copy ribosomal marker genes (in RPKM). One-way ANOVAs were used to test for significant differences in the abundance of metabolic marker genes between shallow and deep sediments, and *p* values were adjusted for false discovery rates with the base R function *p.adjust*. Given that variable completeness of MAGs leads to underestimation of genes present in the microbial group, the percentage occurrence of metabolic marker genes for each order was normalized to inferred genome completeness of the MAGs from each order. Raw data and normalized data are provided in Table S9. To support functional prediction using metabolic marker genes, we further analyzed the completeness of corresponding pathways in MAGs using METABOLIC v.4.0 [78]. A pathway is considered present if over 70% of genes involved were detected (Table S9).

### Phylogenetic analysis

Phylogenetic trees were constructed to verify the presence of key genes involved in energy conservation and carbon fixation in the permeable sediment MAGs and to determine which lineages were present. Trees were constructed for subunits of dissimilatory sulfite reductase (DsrA), sulfide-quinone oxidoreductase (Sqr), flavocytochrome *c* sulfide dehydrogenase (FCC), thiohydrolase (SoxB), acetyl-CoA synthase (AcsB), form I carbon monoxide dehydrogenase (CoxL), group 1 [NiFe]-hydrogenases (large subunit), group 3 [NiFe]-hydrogenases (large subunit), two nitrate reductases (NarG, NapA), three nitrite reductases (NirS, NirK, NrfA), nitric oxide reductase (NorB), nitrous oxide reductase (NosZ), decaheme iron reductase (MtrB), reductive dehalogenase (RdhA), fumarate reductase (FrdA), photosystem II (PsbA), energy-converting microbial rhodopsins, and RuBisCO (RbcL). In all cases, protein sequences retrieved by homology-based searches from the MAGs, and for PsbA also from the unbinned contigs, were aligned against a subset of reference sequences from the custom protein databases using ClustalW [79] in MEGA7 [80]. Evolutionary relationships were visualized by constructing maximum-likelihood phylogenetic trees; specifically, initial trees for the heuristic search were obtained automatically by applying Neighbour-Join and BioNJ algorithms to a matrix of pairwise distances estimated using a JTT model, and then selecting the topology with superior log likelihood value. All residues were used and trees were bootstrapped with 50 replicates.

### Biogeochemical experiments

Slurry experiments were performed to investigate the functional capacity of surface and deep intertidal sands. Each slurry comprised a 160 mL serum vial containing 30 g of sieved sand (wet weight) and 70 mL of seawater (filtered on 0.45 µm Whatman membrane filters). The serum vials were sealed with butyl rubber stoppers and Wheaton closed-top seals. Anoxic slurries were used to measure hydrogenogenic fermentation and sulfate reduction in shallow and deep sands collected on November 12, 2018. Briefly, the slurries were purged with high-purity helium and the headspace was amended with 100 ppmv H_2_. Glucose was added to a final concentration of 1 mM for the glucose addition group. All vials were incubated on a shaker (100 rpm) at room temperature (carefully maintained at 21 °C). For H_2_ measurements, a 2 mL subsample was collected from headspace every 24 h and analyzed by gas chromatography. Three independent slurries were measured for each timepoint and treatment condition. To measure the sulfide produced, one serum vial for each of the six timepoints (0, 48, 96, 144, 196, 360 h) was destructively sampled; a total of 8 mL of seawater was extracted from each slurry and filtered for spectrophotometric analysis. Oxic slurries were used to measure aerobic sulfide oxidation in shallow and deep sands collected on December 6, 2018. The serum vials were aerated with lab air and sodium sulfide (Na_2_S.9H_2_O) was added to a final concentration of 500 μM. All vials were incubated on a shaker (100 rpm) at room temperature. To measure the sulfide consumed, one serum vial for each of the six timepoints (1, 2, 4, 8, 24, 48 h) was destructively sampled; a total of 8 mL of seawater was extracted from each slurry and filtered for spectrophotometric analysis. The autoclaved vial was used as the control group to control for the photochemical oxidation of sulfide in aqueous solution. The amount of biogenic sulfide oxidation that occurred between each timepoint was determined by calculating the difference between the treatment and control groups.

### Molecular hydrogen and sulfide measurements

To measure molecular hydrogen (H_2_), 2 mL gas samples extracted during the slurry experiments were injected into a VICI Trace Gas Analyser Model 6K (Valco Instruments Co. Inc., USA) fitted with a pulsed discharge helium ionization detector as previously described [81]. Ultra-pure helium (99.999% pure, AirLiquide) was used as a carrier gas at a pressure of 90 psi. The temperatures of column A (HayeSep DB), column B (Molesieve 5 Å), and the detector were 55 °C, 140 °C and 100 °C respectively. The instrument was calibrated using standards of ultra-pure H_2_ (99.999% pure, AirLiquide) in ultra-pure He. Sulfide concentrations were quantified through the methylene blue method with GBC UV-Visible 918 Spectrophotometer at 670 nm as previously described [82].

### Microcosm experiments

Microcosm experiments were performed to compare how habitat stability and variability affects the community structure of permeable sediments. Surface (0–3 cm) and deep (20–25 cm) intertidal sediments were collected from Middle Park beach on October 9, 2019. They were incubated in slurries comprising a 160 mL serum vial containing 30 g of sieved sand (wet weight) and 70 mL of seawater (filtered on 0.45 µm Whatman membrane filters). The vials were sealed with butyl rubber stoppers and Wheaton closed-top seals. All vials were incubated on a shaker (100 rpm) at room temperature. Three different treatments were applied for both surface and deep. For the light oxic slurries, vials were aerated daily with laboratory air and continuously exposed to 60 μmol photons m^−2^ s^−1^. For the dark anoxic slurries, vials were purged with high-purity nitrogen gas and covered with aluminum foil. For the oxic-anoxic transition slurries, vials were transferred between light oxic to dark anoxic conditions every 24 h. All incubations were performed in triplicate. DNA was extracted from the original sediments (control group) and each slurry after 14 days of incubation. Community composition was determined by 16S rRNA gene amplicon sequencing as described above, with a total of 19,572 ASVs retained (Table S11).

## Results

### Habitat generalists dominate permeable sediments, but coexist with depth-restricted specialists

**Fig. 1:**
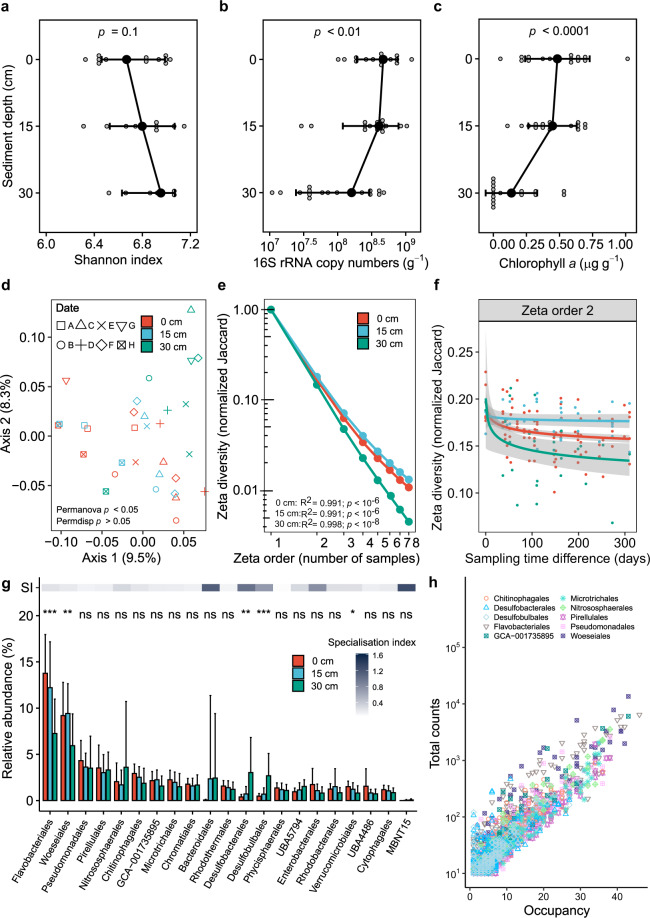
Composition, diversity, and distribution of bacterial and archaeal taxa in permeable sediments. Results are shown based on 16S rRNA gene sequencing for 48 samples covering two tidal zones (intertidal, subtidal), three sediment depths (0–3 cm, 13–17 cm, 27–30 cm), and eight sampling times (between Oct 2016 and Oct 2017). Variations in (**a**) Shannon index (alpha diversity), (**b**) 16S rRNA gene copy number, and (**c**) chlorophyll *a* concentration are shown with depth; error bars show standard deviations of the mean and significance was tested using one-way ANOVAs. **d** Principal coordinates analysis (PCoA) plot visualizing pairwise dissimilatory (beta diversity) of communities using weighted UniFrac. Colors and shapes differentiate samples by sediment depth and sampling date respectively. **e** Zeta decline showing how the average number of ASVs shared between sites decreases as more samples are added. Zeta diversity was calculated for each sediment depth and was normalized to account for differences in richness between samples (Jaccard normalization). *P* values and *R*^2^ values are shown for a power law regression for each depth. **f** Zeta decay showing how the average number of ASVs shared between pairs of sites decreases with sampling date at each sediment depth. Power law regression curves and 95% confidence intervals are shown. **g** Relative abundance of the 20 most abundant orders within the sediments, as well as binned candidate lineage MBNT15; error bars show standard deviations of the mean and significance was tested using linear regression analyses with depth treated as a continuous variable (*p < 0.05, **p < 0.01, ***p < 0.001. ns *p* > 0.05 (not significant)). The above heatmap shows the specialization index (SI) for each taxon based on the coefficient of variance of their relative abundance across the dataset; SIs below the community-wide SI means of 0.64 (order level) indicate relative habitat generalists, SIs above these means indicate relative habitat specialists. **h** Occupancy-abundance relationship of ASVs for ten of the most abundant orders. Each dot shows the abundance (based on total sequence counts) and occupancy (i.e., number of samples present) for each ASV. Further analyses of beta diversity, zeta diversity, occupancy-abundance relationships, specialization indices, and genus- and family-level distributions are provided in the supplementary figures and tables.

We used the 16S rRNA gene as a marker to profile the diversity, abundance, and composition of the bacterial and archaeal communities in permeable sediments. Forty-eight sand samples were profiled that were collected from intertidal and subtidal zones at three different depths (shallow: 0–3 cm, intermediate: 14–17 cm, deep: 27–30 cm) and across eight different dates over the course of a year (Table S2). Alpha diversity indices indicated that the sands support the co-existence of diverse microorganisms (Fig. 1a); Shannon indices were high across the samples (6.78 ± 0.31), with no significant differences observed with sediment depth, tidal zone, or sampling time (Fig. S1**;** Table S4). However, there was a significant decrease in bacterial abundance with depth (inferred from 16S rRNA gene copy number by qPCR) across the samples (Fig. 1b). This correlated with the transition from the mixing layer (above 20 cm) to the sustained aphotic anoxic zone (below 20 cm), as indicated by a sharp decrease in chlorophyll *a* abundance (Fig. 1c) and an increase in acid-volatile sulfide concentrations (from below detection limits to 0.16 µmol g^−1^).

At the amplicon sequence variant (ASV) level, we observed mild differentiation in community composition between samples. Based on pairwise comparisons (weighted UniFrac), community composition was moderately correlated with sediment depth (*R*^2^ = 0.29) and weakly correlated with sampling date (*R*^2^ = 0.08) (Fig. 1d; Table S4). In PCoA and NMDS ordinations (Fig. 1d; Fig. S2), there was tight clustering and insignificant differences in community composition between shallow and intermediate sands (*p* = 0.961), whereas the deep communities were distinct (*p* = 0.008) and showed greater temporal variation. Based on zeta diversity analysis (i.e., average number of ASVs shared across multiple samples [83, 84]), niche differentiation processes were predicted to be dominant drivers of community assembly at all depths (Table S4). Sediments in the mixing zone showed consistently higher zeta diversity (i.e., more taxa shared between samples) than deeper sediments, particularly when increasing number of samples were considered (Fig. 1e; Table S4). There was minimal variation in number of shared taxa over time for the shallow and intermediate samples, based on both pairwise (Fig. 1f) and multisite comparisons (Fig. S3), suggesting community members in the mixing zone are relatively resilient to disturbance. In contrast, community composition in the deep sands exhibited a steep temporal decay (Fig. 1f; Fig. S3), indicating rapid taxonomic turnover. In combination, these results support the theory that disturbance increases homogenization between communities.

Community profiling indicated that the sediment communities were dominated by habitat generalists (Table S2 & S4). In line with previous observations [43], the most abundant orders were Woeseiales and Flavobacteriales (respectively comprising 8.2 ± 3.7% and 11.1 ± 5.0% of the total community), both of which were detected across all samples (Fig. 1g; Fig. S4). Concordant findings were also made at the family, genus, and ASV levels. UBA1844 (Woeseiales) and *Eudoraea* (Flavobacteriales) were the most abundant taxonomically assigned genera detected (Fig. S5). Likewise, the 11 most abundant ASVs and ten most prevalent ASVs all affiliated with these two orders (including UBA1844 and *Eudoraea*) and uncultured gammaproteobacterial lineage GCA-1735895 (Fig. 1h; Fig. S4 & S6). Nevertheless, within each of these orders, there were also multiple narrowly distributed and less abundant ASVs (Fig. 1h; Fig. S6). Various ASVs within the orders Pseudomonadales, Pirellulales, Microtrichales, Chitinophagales, and Nitrososphaerales were also highly abundant and prevalent (Fig. 1g & 1h; Fig. S4). We calculated specialization indices for each order, family, and genus, based on the coefficient of variance of their relative abundance across samples as previously described [85], to predict whether they have strong or weak habitat preferences (Table S5). The specialization indices of the eight aforementioned orders were two-to fivefold lower than the mean for the community, indicating they are habitat generalists. The perpetual abundance of these lineages suggests they can withstand large variations in physicochemical conditions and resource availability in these sands. Note the 16S rRNA gene analysis used a set of primers known to introduce some bias in community profiling [51] and have recently been superseded by new primer sets [86]. However, these bacterial groups were also the most abundant in metagenomes (Table S6), based on community profiling using a conserved single-copy ribosomal protein gene (Table S7 & Fig. S7).

The specialization indices of some lineages were above the mean for the community, suggesting they are relative habitat specialists (Table S5). Most notably, Desulfobacterales, Desulfobulbales, and Bacteroidales greatly increased in relative abundance with depth (Fig. 1g) and drove much of the community differentiation observed between the deep samples compared to those in the mixing zone (Fig. 1d). This indicates that the anoxic conditions of these sediments have selected for expansion of anaerobic specialists, including sulfate-reducing bacteria. However, their relative abundance greatly varied within the community across sampling dates; for example, while Desulfobacterales and Desulfobulbales comprised up to 15% and 6% of the community in deep intertidal sediments, they were both absent from such sediments at the penultimate sampling point. Likewise, while the candidate lineage MBNT15 was generally rare in the sediments (Table S2; Fig. S4), it became transiently abundant in deeper samples based on amplicon (Fig. 1f) and metagenome (Fig. S5) sequencing data. While the reason for these differences is unclear, it is possible that these more specialist taxa are relatively sensitive to the disturbance events (e.g., oxygenation) that still occasionally affect deeper sediments, in contrast to the habitat generalists they coexist with. Alternatively, temporal variations in habitat conditions (e.g., due to seasonality) may select specialist taxa in hydrodynamically stable deep sediments, as inferred by a steeper temporal decay of zeta diversity (Fig. S3). Aerobic specialists (i.e., taxa with high specialization indices that predominated in the mixing zones) were less abundant, and included genera within the order Enterobacterales (e.g., *Psychrosphaera*) (Fig. 1f; Table S5). Concordant findings were observed at finer taxonomic resolution; ASVs from the five most abundant specialist orders had significantly lower average and maximum occupancies (i.e., proportion of sampled sites in which they were detected) than those from the most abundant habitat generalist orders (Fig. S4 & S6).

### Metabolic flexibility differentiates habitat generalists and specialists

**Fig. 2:**
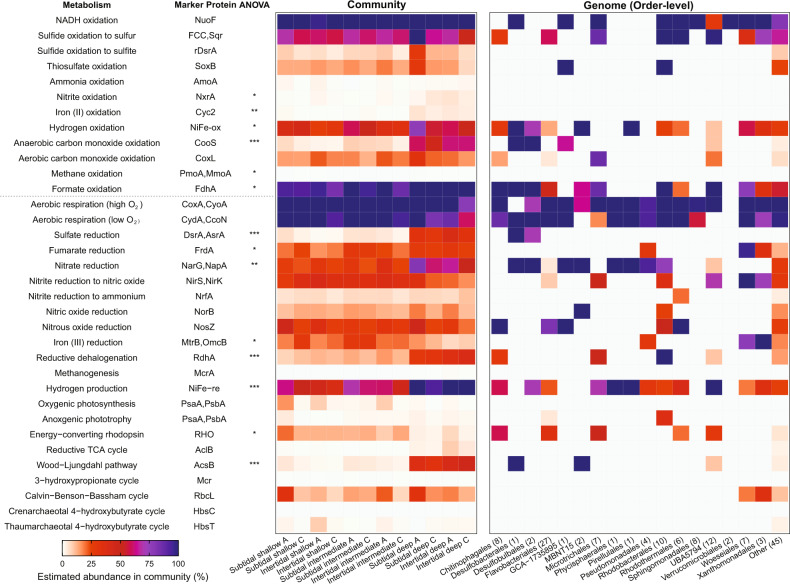
Metabolic capacity of microbial communities in permeable sediments. Homology-based searches were used to detect key metabolic genes in 12 metagenomes (Table S6 & S10) and 147 derived metagenome-assembled genomes (MAGs; Table S10). The left columns show the proportion of community members in each metagenome predicted to encode each gene based on the short reads; hits were normalized to gene length and single-copy ribosomal marker genes. Hits were summed for each process where more than one gene was searched for (up to 100%), with the exception of oxygenic photosynthesis where PsaA and PsbA hits were averaged (reflecting both genes are required for this process to occur). The right columns show the proportion of MAGs estimated to encode each gene, with results shown by order; hits are normalized based on estimated genome completeness of each order. Metabolic marker genes involved in the oxidation of electron donors (top rows), reduction of electron acceptors (middle rows), and fixation of inorganic carbon (bottom rows) are shown. NiFe-ox and NiFe-re denotes [NiFe]-hydrogenases involved in H_2_ oxidation (group 1 and 2a) and H_2_ production (group 3 and 4) respectively. One-way ANOVAs were used to test whether there were significant differences in relative abundance of genes between depths (*p < 0.05, **p < 0.01, ***p < 0.001, blank = not significant between shallow and deep sediments).

We used genome-resolved metagenomics to gain an insight into the metabolic traits of the habitat generalists and specialists detected. Sequencing, assembly, and binning of metagenomes of intertidal and subtidal sands from each sediment depth (Table S6 & S8) yielded 38 high-quality and 97 medium-quality MAGs [71] (Table S9). We additionally reanalyzed the 12 MAGs that we previously reported from this study site [43]. Together, the resultant genomes span 13 phyla and 43 orders, including 17 of the 20 most abundant orders detected by 16S rRNA gene profiling (Fig. 1). We profiled the abundance of 51 metabolic marker genes in the short reads (Table S8), derived MAGs (Table S9), and unbinned contigs (Table S10) to gain insights into the functional capabilities of the habitat generalists and specialists (Fig. 2). Based on the short reads, the percentage of total bacterial cells that perform each metabolic process was calculated based on the ratio of metabolic marker genes to universal single-copy ribosomal protein marker genes (both in RPKM) (Table S8).

**Fig. 3:**
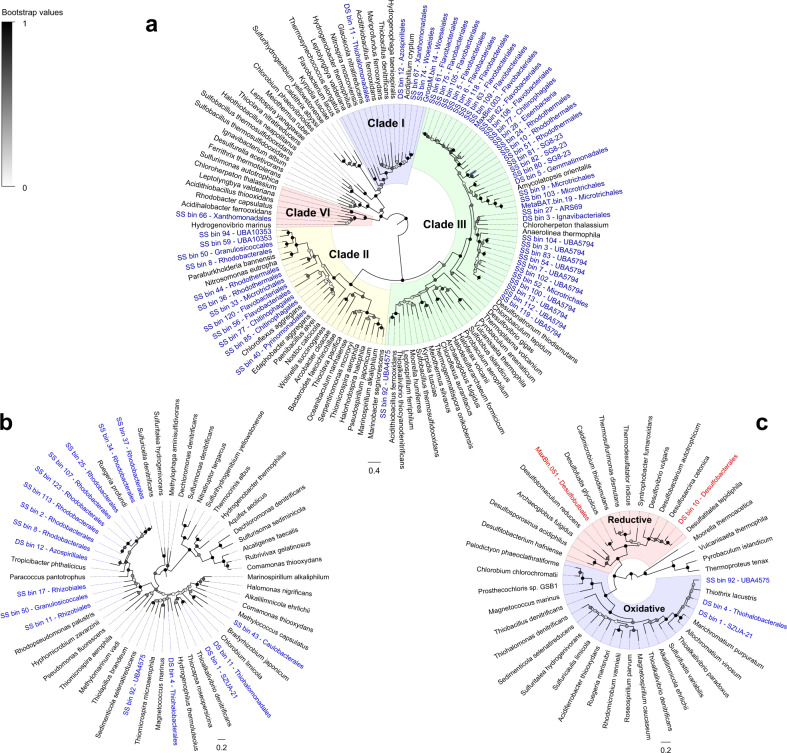
Phylogenetic trees of genes mediating sulfur cycling. Maximum-likelihood phylogenetic trees are shown for (**a**) sulfide-quinone oxidoreductase (Sqr), (**b**) flavocytochrome *c* sulfide dehydrogenase (FCC), and (**c**) dissimilatory sulfite reductase A subunit (DsrA). The tree shows sequences from permeable sediment metagenome-assembled genomes (colored) alongside representative reference sequences (black). The trees were constructed using the JTT matrix-based model, used all sites, and were midpoint-rooted. The four Sqr clades and two DsrA clades present in the MAGs are differentiated shaded. Note Sqr, FCC, and the oxidative clade of DsrA (rDsrA; encompassing Proteobacteria MAGs) are known to mediate aerobic sulfide oxidation (MAGs colored in blue), whereas the reductive clade of DsrA (encompassing Desulfobacterota MAGs) mediate anaerobic sulfite reduction (MAGs colored in red). Node junctions represent bootstrap support from 50 replicates. Full linear trees with accession numbers are provided in Fig. S8 (Sqr), Fig. S9 (FCC), and Fig. S11 (DsrA).

Most community members are predicted to be aerobic heterotrophs capable of using organic and inorganic energy sources. Based on short reads (Table S8) and assemblies (Table S10), most bacteria encoded enzymes for sulfide or thiosulfate oxidation, i.e., sulfide-quinone oxidoreductase (Sqr, 54% of total community), flavocytochrome *c* sulfide dehydrogenase (FCC, 12%), reverse dissimilatory sulfite reductase (rDsrA, 9%), and thiosulfohydrolase (SoxB, 16%) (Fig. 2). Concordantly, a similar proportion of the MAGs encoded these enzymes (Fig. 2**;** Table S6) and phylogenetic trees confirmed all binned sequences affiliated with canonical clades (Fig. 3**;** Fig. S8–S11). Diverse Sqr sequences were detected, including in Woeseiales, Flavobacteriales, Rhodobacterales, and Microtrichales MAGs (Fig. 2**;** Table S9), with particularly high abundance of the type III Sqr clade (Fig. 3a) known to support sulfide-dependent growth [87, 88]. Also widespread were the genes for consumption of carbon monoxide (CoxL, 19%; Fig. S12) and hydrogen gas (group 1 and 2 [NiFe]-hydrogenases, 47%; Fig. S13). Most bacteria also appear to have a large capacity to withstand variations in electron acceptor availability. In addition to encoding terminal oxidases for aerobic respiration (Fig. 2), many are predicted to mediate stepwise denitrification through nitrate (NarG and NapA, 40%; Figs. S14 & S15), nitrite (NirS and NirK, 32%; Figs. S16 & S17), nitric oxide (NorB, 14%; Fig. S18), and nitrous oxide (NosZ, 31%; Fig. S19), with fewer mediating dissimilatory nitrate reduction to ammonium (DNRA *via* NrfA, 7%; Fig. S20) (Fig. 2). As we previously reported [43], hydrogenotrophic sulfur reduction (group 1e [NiFe]-hydrogenases, 17%; Fig. S13) and facultative hydrogenogenic fermentation (group 3 [NiFe]-hydrogenases, 61%; Fig. S21) are also common. Diverse community members were also capable of reducing other compounds (Table S8 & S9), such as ferric iron (MtrB, 20%; Fig. S22) and organohalides (RdhA, 19%; Fig. S23). By contrast, few are predicted to mediate the specialist traits of ammonia, iron, nitrite, or methane oxidation, methanogenesis, acetogenesis, and, in the mixing zone, sulfate reduction (Fig. 2; Tables S8–S10).

Further analysis of the reconstructed genomes revealed that the most prevalent taxa are highly metabolically flexible (Fig. 2; Table S9). The Woeseiaceae MAGs, representing one of the most abundant and prevalent families in the sediments, encode enzymes for aerobic heterotrophy, sulfide oxidation, hydrogenotrophic sulfur reduction, denitrification, FrdA (Fig. S24), iron reduction, hydrogenogenic fermentation, and for one MAG, chemosynthetic carbon fixation (Fig. S25). Flavobacteriaceae are similarly flexible, for example with *Eudoraea* MAGs encoding genes to harness energy from organic carbon, sulfide, hydrogen, and sunlight via proteorhodopsin (Fig. S26), as well as switching between aerobic respiration, anaerobic respiration, and fermentation. Other inferred habitat generalists, including within highly abundant orders Pseudomonadales, Pirellulales, Microtrichales, Rhodothermales, and GCA-1735895 (Fig. 1g), are also predicted to be able to use multiple energy sources and electron acceptors in these sediments (Fig. 2). Altogether, these data suggest that most community members can accommodate environmental fluctuations in the availability of oxygen and other electron acceptors by switching between different respiratory and fermentative processes. Moreover, they can take advantage of a wide range of organic and inorganic energy sources that are likely to be abundant in these sediments. While most of the bacteria in the sediments were predicted to be flexible, we detected no lithotrophy or anaerobic respiration pathways across multiple near-complete Sphingomonadales and Verrucomicrobiales MAGs (Table S9), suggesting they are constrained to an aerobic organotrophic lifestyle, in line with their habitat preference for surface sands (Fig. 1g; Table S2). We also annotated the MAGs using the more extensive, but less curated, reference databases provided with the tool METABOLIC [78]; this produced largely concordant findings, confirming high completeness of the pathways identified through our marker gene approach (Table S9).

The metagenomes also provide insights into the metabolic capabilities of community members with more restricted distributions (i.e., relative habitat specialists). Whereas the relative abundance of many genes associated with habitat generalists (e.g., sulfide oxidation) did not change with depth, there was a significant tenfold increase in the relative abundance (*p* < 0.001) of the marker genes for dissimilatory sulfate reduction (DsrA) (Fig. 3c; Fig. S10) and the Wood-Ljungdahl pathway (AcsB, CooS) (Fig. S27) in the metagenomes of deep sands compared to shallow and intermediate sands. This strongly correlates with the increased abundance of sulfate-reducing bacteria from the orders Desulfobulbales and Desulfobacterales at these depths (Fig. 1f) that encode these genes (Fig. 2). These bacteria are likely able to thrive in this niche by coupling the oxidation of the fermentative endproducts hydrogen (*via* group 1b and 1c [NiFe]-hydrogenases; Fig. S13) and acetate (through the oxidative Wood-Ljungdahl pathway; Fig. S27) to sulfate reduction. As shown by the metabolic heatmap in Fig. 2 and phylogenetic trees of Fig. 3, the genes for the inferred specialist process of sulfate reduction were far less abundant and less taxonomically widespread than those for sulfide oxidation. These sulfate-reducing orders nevertheless possess some respiratory flexibility, including the ability to use nitrate (Fig. S15) and organohalides (Fig. S23), suggesting they can accommodate some changes in resource availability. They also possess cytochrome *bd* and cytochrome *cbb*_3_ oxidases that can scavenge trace levels of oxygen (Fig. 2); however, given previous reports that terminal oxidases support oxygen detoxification rather than aerobic growth in sulfate-reducing bacteria, these bacteria are likely to be inhibited rather than stimulated by oxygen in contrast to the facultative anaerobes that they coexist with [89, 90]. Similarly, genome annotations based on homology-based searches and METABOLIC profiling indicate MBNT15 bacteria are obligate anaerobes that couple H_2_ and acetate oxidation to nitrate reduction (Fig. 2; Table S9). Thus, these members of the Desulfobacterales, Desulfobulbales, and MBNT15 appear to be relative habitat specialists that thrive in anoxic deep sediments, but lack the metabolic capabilities to compete in transiently oxygenated surface sediments.

### Metabolic processes associated with habitat generalists and specialists show depth variations in permeable sediments

**Fig. 4:**
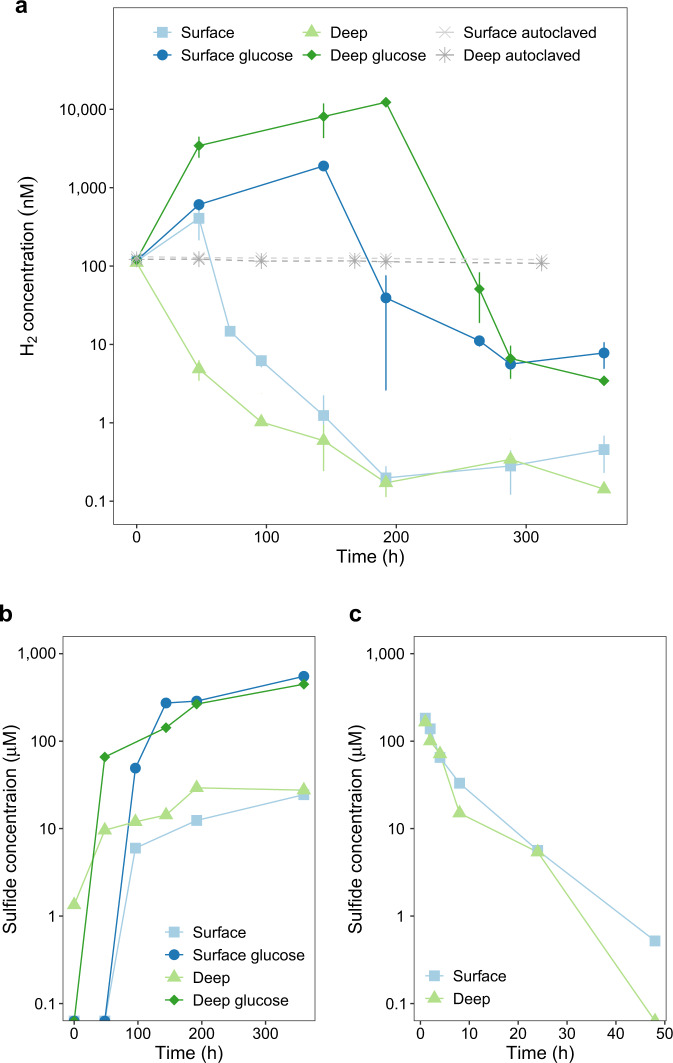
Metabolic activities of microbial communities in permeable sediments. **a** & **b** Capacity of sands to mediate hydrogenogenic fermentation and hydrogenotrophic sulfate reduction under anoxic conditions. Shallow and deep sediments were incubated in nitrogen-purged slurries in the presence of 100 ppmv H_2_ and, for spiked samples, 1 mM glucose. Changes in (**a**) H_2_ concentration and (**b**) sulfide concentration were measured during the experiment. For H_2_ measurements, error bars show standard deviations for three independent slurries. **c** Capacity of oxic sands to mediate sulfide oxidation. Shallow and deep sediments were each incubated under oxic conditions in six independent slurries amended with 200 µM Na_2_S.9H_2_O. Changes in sulfide concentration were measured during the timecourse, with one serum vial sacrificed per timepoint.

The above findings suggest that several alternative metabolic pathways, such as sulfide oxidation and hydrogenogenic fermentation, allow habitat generalists to adapt to changes in resource availability. The relative abundance of community members that mediate these processes, as well as the metabolic genes that they encode, is similar across depth (Figs. 1g & 2). Thus, it can be expected that these processes occur in both shallow and deep sediments. To test this, we first measured rates of sulfide oxidation in intertidal sediments spiked with sodium sulfide under oxic conditions. Sulfide was rapidly consumed in a first-order kinetic process to below detection limits in both shallow and deep sediments (Fig. 4c). We also measured hydrogenogenic fermentation in sands under anoxic conditions; glucose addition stimulated rapid accumulation of molecular hydrogen to micromolar levels in both surface and deep sands (Fig. 4a).

In contrast, the community and metagenomic data indicate that sulfate reducers are habitat specialists that preferentially reside in the deeper sediments. To verify this, we measured rates of hydrogenotrophic sulfate reduction in anoxic H_2_-supplemented surface and deep intertidal sediments. As anticipated given the abundance of hydrogenotrophic sulfate reducers (Fig. 1f) and *dsrA* genes (Fig. 2), the microbial communities in deep sediments consumed most H_2_ within 48 h (Fig. 4a), concomitant with accumulation of 10 µM sulfide (Fig. 4b). In contrast, fermentation and respiration became uncoupled in surface sediments following the onset of anoxia; rates of fermentation initially exceeded respiration, resulting in net H_2_ accumulation and no detectable sulfide production within 48 h. This is in line with our previous in situ and ex situ observations that fermentation dominates carbon mineralization in well-mixed permeable sediments irrespective of the availability of anaerobic electron acceptors [43, 45]. Hydrogenotrophic sulfate reduction only became dominant after prolonged incubations under anoxia (Fig. 4a & 4b), likely due to growth of sulfate-reducing bacteria under these stable conditions.

### Metabolically flexible bacteria outcompete specialists during simulated disturbance events

**Fig. 5:**
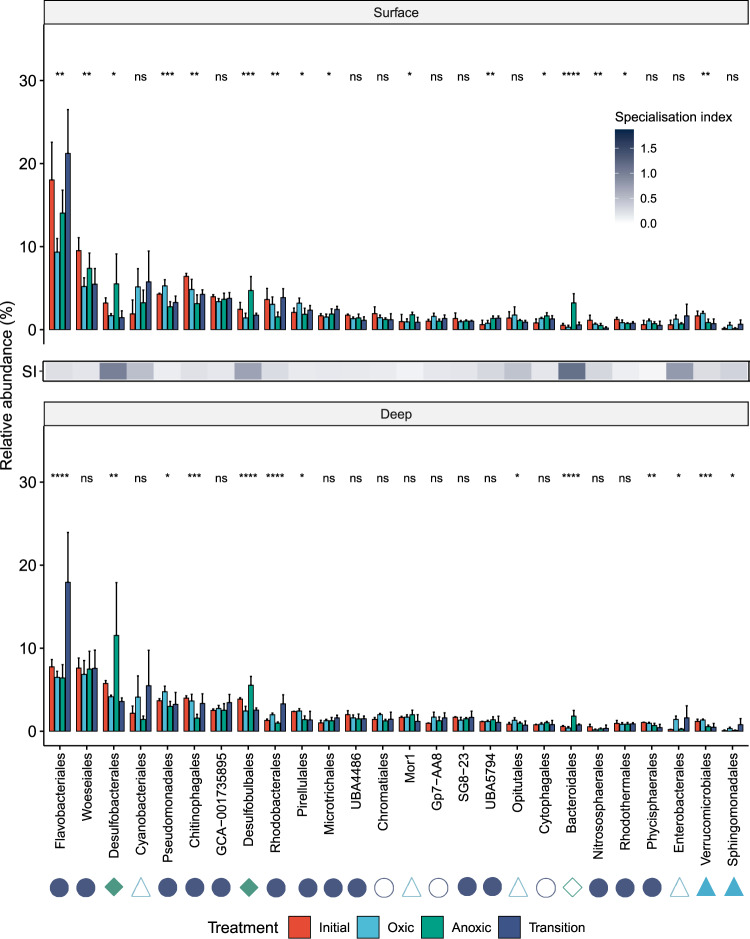
Responses of different orders to simulated environmental disturbance. The relative abundance of the 26 most abundant microbial orders from surface (top) and deep (bottom) sands is depicted with red bars. The changes of their relative abundance is shown after sands were incubated in slurries for 2 weeks in one of three conditions: continual light oxic conditions (light blue bars), continual dark anoxic conditions (green bars), or disrupted conditions (dark blue bars) in which slurries were shifted between light oxic and dark anoxic conditions every 24 h. Error bars show standard deviations of the mean and significance was tested using one-way ANOVAs (*p < 0.05, **p < 0.01, ***p < 0.001, ****p < 0.0001, ns *p* > 0.05 (not significant)). The heatmap depicts the specialization index (SI) for each taxon based on the coefficient of variance of their relative abundance across the longitudinal study depicted in Fig. 1; SIs below the community-wide SI means of 0.64 (order level) indicate relative habitat generalists, SIs above these means indicate relative habitat specialists. Shapes next to taxon names predict metabolic capabilities of each order based on the obtained MAGs: facultative anaerobes (dark blue circles), obligate aerobes (light blue triangles), and obligate anaerobes (green diamonds). Given no MAGs were obtained for Cyanobacteriales (chloroplasts), Chromatiales, Mor1, Gp7-AA8, Opitulales, Cytophagales, Bacteroidales, and Enterobacterales, metabolic capabilities are predicted based on their cultured closest relatives (open shapes). Results are shown at family and genus levels in Fig. S28.

In combination, the community, metagenomic, and biogeochemical profiles suggest that metabolic flexibility facilitates habitat generalism of microorganisms in permeable sediments. We performed a manipulative incubation experiment to test whether this inference is valid. Samples collected from shallow and deep intertidal sediments were incubated for 14 days under one of three conditions: continual light oxic conditions, continual dark anoxic conditions, and disturbed conditions (24 h cycles between light oxic and dark anoxic conditions). It should be noted that these microcosms do not fully capture the conditions and complexity of the natural ecosystem, and some selection may have been introduced due to temperature differences, physical shaking, and bottle effects. Nevertheless, we observed significant changes in the relative abundance of many key taxa previously highlighted in the analysis of community composition (Fig. 1) and function (Fig. 2) between the three incubation conditions (Fig. 5; Table S11).

Although most taxa exhibited significant changes in relative abundance during the incubations, those predicted to be metabolically flexible were perpetually abundant. Taxa inferred to be metabolically flexible habitat generalists were dominant in all samples, and were most abundant compared to taxa inferred to be metabolically constrained habitat specialists in the original samples and disturbed incubations (Fig. 5). Modest changes in the relative abundance of Woeseiales, Microtrichales, Rhodothermales, and GCA-1735895 lineages were observed between the time of sampling and following two weeks of incubations. We also monitored the patterns of lineages predicted to be aerobic specialists (from orders Enterobacterales, Verrucomicrobiales, and Sphingomonadales) and anaerobic specialists (from orders Desulfobacterales, Desulfobulbales, and Bacteroidales) based on their constrained metabolic capabilities (Fig. 2) and high specialization indices (Fig. 1g**;** Table S5). The three lineages of inferred aerobic specialists, while always relatively minor constituents of the community, were most abundant in oxic incubations (total 3.3% relative abundance) and least in anoxic sediments (1.2%). Inferred anaerobic specialists showed the opposite pattern. They bloomed to one-sixth of the community in the anoxic incubations (16%), with large enrichments in the sulfate-reducing families Desulfocapsaceae, Desulfosarcinaceae, and Desulfobacteraceae, but declined during oxygen exposure (5.6%) (Fig. 5; Fig. S28). Under stable anoxic conditions, these anaerobic specialists likely rapidly mobilize available resources through their sulfate reduction and fermentation pathways. The relative abundance of most aerobic and anaerobic specialists declined in the disturbed slurries compared to the original samples, suggesting cell death (Fig. 5). An exception was Alteromonadaceae (Enterobacterales) (Fig. 5; Fig. S28), potentially reflecting that this family symbiotically associates with diatoms in permeable sediments [43, 91].

Remarkably, some taxa thrived in response to disturbance. Flavobacteriales sampled from deep sediments increased in relative abundance by 1.9-fold in the disturbed incubations (Fig. 5), largely driven by expansions of the genus *Eudoraea* (Fig. S28). Based on the metabolic capabilities of the three MAGs from this genus (Table S9), such bacteria may take advantage of necromass released during oxic-anoxic transitions by switching between aerobic respiration and hydrogenogenic fermentation pathways. Likewise, there were significant enrichments in the two dominant lineages harboring photosystems (Fig. S29), namely photoheterotrophic Rhodobacteraceae and photoautotrophic diatoms (detected by chloroplast 16S rRNA gene sequences) (Table S11; Fig. 5). These taxa likely benefit from the increased light availability under both the light oxic and disturbed conditions compared to natural sediments, but also possess genes that enable adaptation to dark anoxic conditions (Table S9). Such flexibility is apparent from the diverse repertoire of the Rhodobacteraceae lineages *Sulfitobacter* and *Silicimonas*, which encode the determinants of aerobic anoxygenic photoheterotrophy [92] together with those for sulfur compound oxidation, reductive dehalogenation, and variably denitrification (Fig. 2; Table S9). These inferences are also supported by previous studies inferring benthic diatoms survive dark anoxic conditions through nitrate respiration [93] and microbiota-mediated hydrogenogenic fermentation [43, 45]. Although this experiment generally substantiated metagenome-based inferences, a few taxa behaved contrary to predictions. Most notably, Chitinophagales significantly decreased under anoxic conditions despite being predicted to be habitat generalists based on specialization index (Fig. 1g; Table S5) and harboring genes for hydrogenogenic fermentation (Fig. S20; Table S9), suggesting members of this order either cannot survive in these conditions or are outcompeted by more efficient anaerobes; these observations are nevertheless consistent with the decrease in the relative abundance of this order with depth (Fig. 1f).

## Discussion

In combination, these results provide multifaceted evidence that environmental disturbance influences distributions of microbial habitat generalists and specialists. The microbial communities in the mixing zone of permeable sediments experience frequent but irregular spatiotemporal variations in oxygen, sunlight, nutrients, and redox state [30]. Based on ecological theory, it would be expected that these variations would differentially affect generalists and specialists [1, 5]. For the specialists, these changes would promote continual cycles of growth and death as conditions alternate between favorable and unfavorable. In contrast, generalists are expected to maintain more stable populations given they are more adaptable to environmental change. We observed that habitat generalists are indeed more competitive in these environments. Large and stable populations of ASVs from orders such as Woeseiales, Flavobacteriales, and Pseudomonadales were present in both the mixing and deep layers of the sampled sediments across sampling times, and were enriched under simulated disturbance conditions in the manipulative incubations. Thus, in line with observations for macroorganisms, environmental disturbance appears to favor bacterial habitat generalists and promote some degree of homogenization of composition between microbial communities.

Some relative habitat specialists nevertheless coexist with such generalists in these environments. Numerous taxa were detected with low occupancies and abundances, several of which bloomed under favorable conditions, most notably MBNT15. The manipulative incubation experiments confirmed that these inferred specialists were only enriched under more stable conditions (light oxic for aerobes, dark anoxic for anaerobes). Most notably, Desulfobacterales were the most abundant order in deep sediments at certain sampling times and during prolonged dark anoxic incubations, reflecting that sulfate-reducing bacteria thrive in stable hydrogen- and sulfate-rich environments. These taxa and other anaerobic specialists nevertheless exhibited sharp variations in relative abundance across the sampling dates, as well as significant declines under oxic and disturbed incubations. Consistent with ecological theory, this suggests that such habitat specialists are sensitive to the disturbances that define the mixing zone and occasionally affect deeper sands, whereas the generalists that they coexist with are more adaptable. More sampling is required across various spatial and temporal scales to resolve the physicochemical pressures and biological interactions that drive these differences, as well as resolve seasonal changes in community composition. However, it is probable that oxygen availability is the most significant factor that influences composition, for example through causing poisoning of strictly anaerobic habitat specialists or by allowing habitat generalists able to switch between aerobic and anaerobic growth to outcompete strictly anaerobic or aerobic specialists [43]. The relative stability of deeper sediments may also promote formation of physicochemically distinct microenvironments, which would be ideally suited for certain habitat specialists, that would be lost during occasional mixing events.

In turn, our study lends strong support to the hypothesis that microbial habitat generalists and specialists have distinct metabolic capabilities. Based on the reconstructed genomes, the habitat generalists in the community possess much metabolic versatility. Most notably, the Woeseiaceae lineages that dominate these sands are particularly versatile, given their predicted use of a wide spectrum of electron donors (organic carbon, sulfide, hydrogen), oxidants (oxygen, nitrite, fumarate, sulfur, fermentation), and carbon sources (heterotrophy, autotrophy). Flavobacteriaceae and Rhodobacteraceae lineages have similar metabolic breadth, likely facilitating their expansion in response to disturbance. By contrast, relative habitat specialists from the Desulfobacterales and Desulfobulbales are distinguished by their capacity to the use the abundant electron acceptor sulfate, but also their inability to grow by aerobic respiration [89]. These bacteria possess some metabolic flexibility, likely explaining why these orders were detected in low levels even in most surface sediments and oxygenated slurries; indeed, habitat generalism and metabolic flexibility alike should be considered as continuous traits. However, such obligate anaerobes are outcompeted by facultative anaerobes under disturbed conditions. These inferred differences were strongly supported by biogeochemical assays showing that, whereas sulfate reduction is limited to sediments under prolonged anoxia, metabolic traits associated with habitat generalists are active through sediment zones. Further culture-dependent and culture-independent work, however, is required to fully understand the metabolic capabilities of permeable sediment bacteria and their responses to environmental changes.

More broadly, these findings have consequences for understanding the processes controlling co-existence of habitat generalists and specialists. Current macroecological theory suggests that the co-existence of generalists and specialists can be understood via interactions between disturbance, niche breadth, and dispersal ability, whereby disturbance creates extinction-colonization events. As disturbance increases, the habitat niches of specialists become increasingly limited and disconnected. In this scenario, only specialists with high dispersal abilities will be able to colonize available niches and persist in the metacommunity [85]. Our findings may be congruent with this model if it is assumed that microbiota subject to porewater advection and sediment mixing have an inherently high dispersal ability due to the physical mixing of the environment. In our study, specialist taxa never dominated real or simulated sediment environments, but exhibited an increased competitive ability under conducive stable conditions. Such observations are in line with classical r/K selection theory [94, 95], in which specialist taxa are traditionally associated with K-selection and are predicted to dominate in stable environments due to an investment in competitive abilities. Similar studies in environments where there is selection for dispersive traits, such as pelagic ocean microbial communities, could be used to integrate dispersal ability into the understanding of generalist-specialist co-existence dynamics for microbes. In turn, this provides further avenues to investigate the congruence between classical ecological frameworks and microbial communities [96].

These findings also have important implications for how we conceive and model biogeochemical processes. Models describing these processes can either take an organism-centric approach or a systems perspective [97]. In the first case, the presence or absence of a particular organism will determine the process taking place and emphasis is placed on modelling the growth of that organism. In the second case, thermodynamics and physical conditions determine the processes taking place. Biogeochemists typically use the second approach to predict and model sediment processes [46]. Under conditions of continual disturbance, we show that habitat generalists dominate, and the energy conservation pathways that are used (particularly under anaerobic conditions) will not be those predicted from thermodynamics until habitat specialists dominate (such as sulfate reduction). Under disturbed conditions, therefore, community structure and the presence of habitat generalists (the organism-centric view) becomes an important consideration for predicting ecosystem processes. Consistent with this, it has been shown that physicochemical variables are the strongest predictors of microbially driven ecosystem processes, but that microbial community structure can improve these predictions in some cases [98]. Future studies should incorporate disturbance as a co-variate when comparing the efficacy of organism and system scale models (both statistical and deterministic).

In summary, we conclude that habitat generalists thrive in the disturbed environments of permeable sediments and generally outcompete specialists. This reflects their greater metabolic flexibility, particularly their capacity to shift between electron acceptors during oxic-anoxic transitions. Relative habitat specialists have narrower niches, but are highly competitive under more stable conditions. These findings are substantiated through community and metagenomic profiling, biogeochemical measurements, and manipulative experiments. Thus, a long-standing ecological theory explaining differential distribution patterns of macroorganisms appears to extend to microorganisms and we provide a mechanistic rationale for these observations. Though further studies are required to extend these findings beyond permeable sediments, it is probable that metabolic flexibility is a key factor governing distributions of generalist and specialist taxa across ecosystems.

## Supplementary information

Supplementary information

Table S1

Table S2

Table S3

Table S4

Table S5

Table S6

Table S7

Table S8

Table S9

Table S10

Table S11

## Data Availability

All amplicon sequencing data, raw metagenomes, and metagenome-assembled genomes were deposited in the NCBI Sequence Read Archive under BioProject PRJNA609151.
