# Supplementary material for: Metabolic flexibility allows bacterial habitat generalists to become dominant in a frequently disturbed ecosystem
Source: ISME J. 2021 May 3;15(10):2986–3004. doi: 10.1038/s41396-021-00988-w (PMC8443593; doi:10.1038/s41396-021-00988-w)

**Supplementary material**

**Table S1 (xlsx).** Tide and weather details for the eight sampling dates.

**Table S2 (xlsx).** Amplicon sequence variant (ASV) counts detected during the *in situ* survey by 16S rRNA gene amplicon sequencing.

**Table S3 (xlsx).** Comparison of ASV taxonomic assignment by GTDB r89 and SILVA v138 releases.

**Table S4 (xlsx).** Measurement and statistical analysis of alpha, beta, and zeta diversity.

**Table S5 (xlsx).** Specialization indices of each order, family, and genus based on their ecological distributions.

**Table S6 (xlsx).** Sequencing and assembly information on metagenomes analyzed for this study.

**Table S7 (xlsx).** Community composition based on metagenomic reads of the single-copy ribosomal marker gene *rplP*.

**Table S8 (xlsx).** Distribution of metabolic genes in metagenome short reads based on homology-based searches and METABOLIC profiling.

**Table S9 (xlsx).** Taxonomic information and metabolic capabilities of the 147 metagenome-assembled genomes analyzed.

**Table S10 (xlsx).** Sequences of metabolic genes in unbinned contigs based on homology-based searches.

**Table S11 (xlsx).** Amplicon sequence variant (ASV) counts detected during the microcosm experiment by 16S rRNA gene amplicon sequencing.

**Figure S1.** Comparison of alpha diversity metrics based on 16S rRNA gene amplicon sequencing results. Shannon index and observed, Chao1, and ACE richness are compared between **(a)** sampling date, **(b)** sediment depth, and **(c)** tidal zone.

**
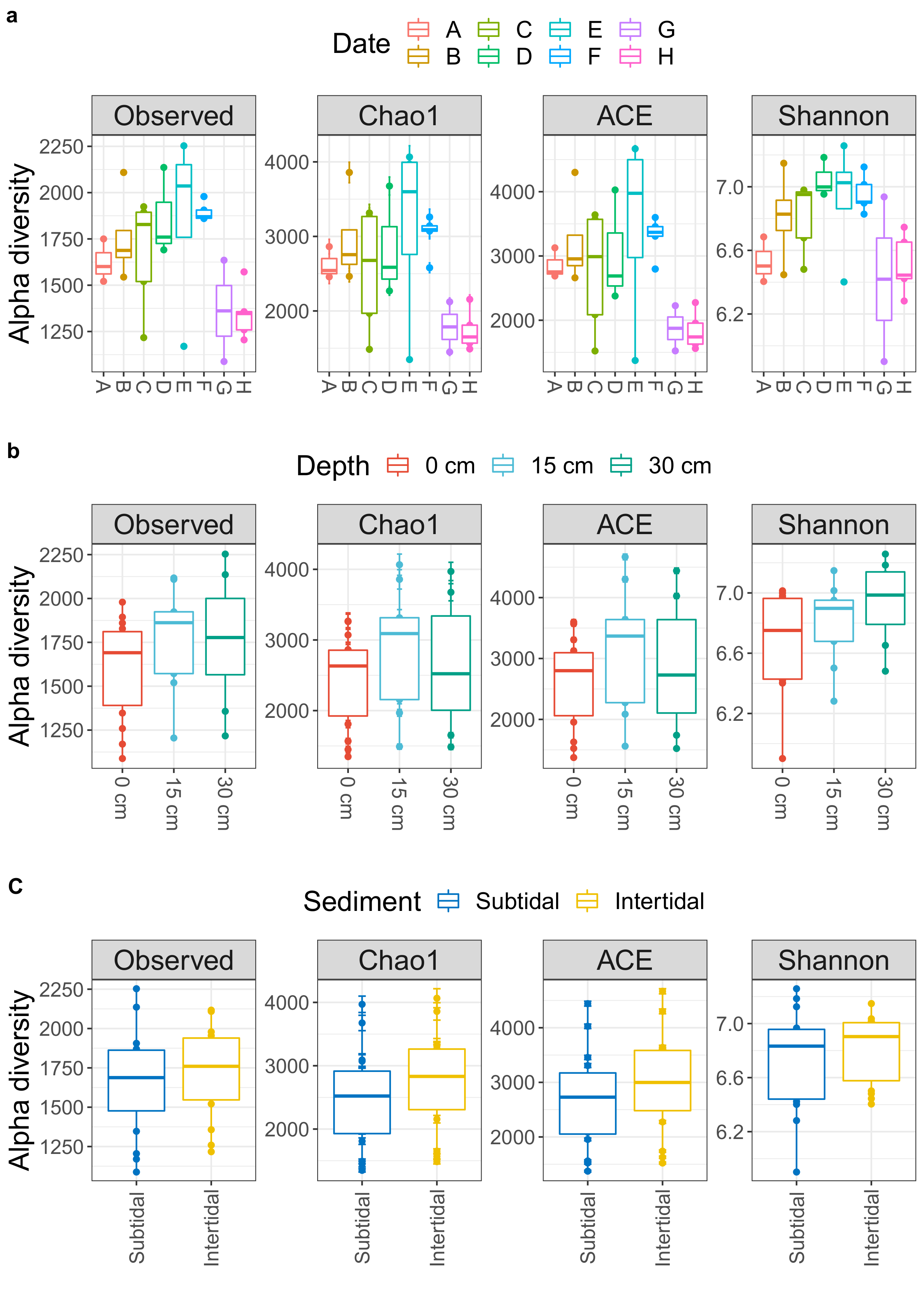
**

**Figure S2.** Visualization of beta diversity (weighted UniFrac) of samples based on nonmetric multidimensional scaling (NMDS). This shows differentiation of community structure, as inferred from 16S rRNA gene amplicon sequencing, based on sediment depth and sampling dates.

**Figure S3.** Temporal decay of zeta diversity based on pairwise and multisite comparisons. This shows the decrease in average number of shared taxa between sites, as inferred from 16S rRNA gene sequencing, with increasing sampling time. Pairwise (zeta order 2) and multisite (zeta orders 3 and 4) comparisons are shown. Each dot represents zeta diversity of each sample combination, regression lines show the power law fit of the zeta diversity, and shadings represent the 95% confidence intervals of the regression for each of the three depths.


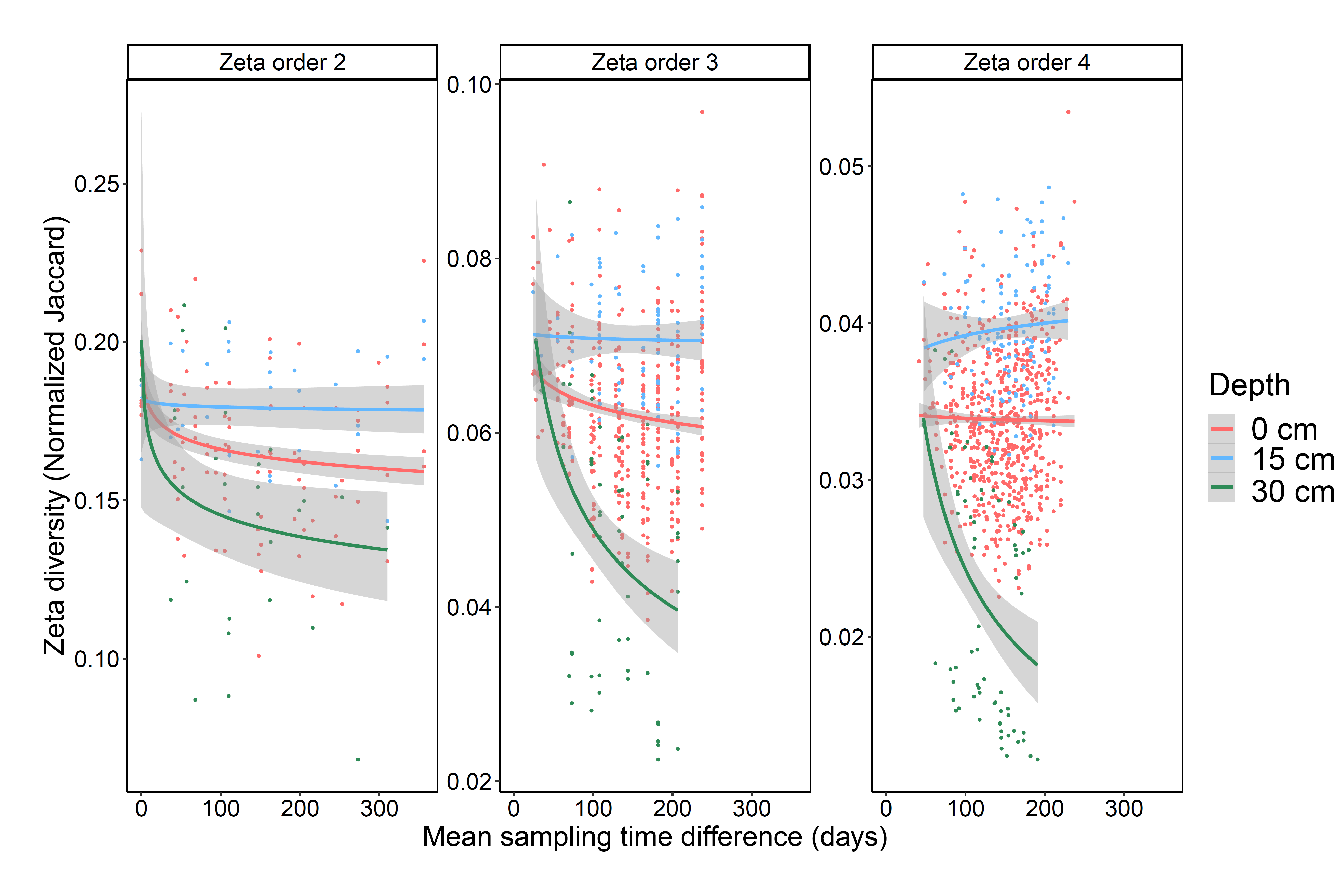


**Figure S4.** Occupancy-abundance relationship of amplicon sequence variants (ASVs) by order. Results are shown for the 20 most abundant orders and MBNT15 based on 16S rRNA gene amplicon sequencing. Each dot shows the average relative abundance of a given ASV and their percentage occupancy across the samples at each of the three depths.


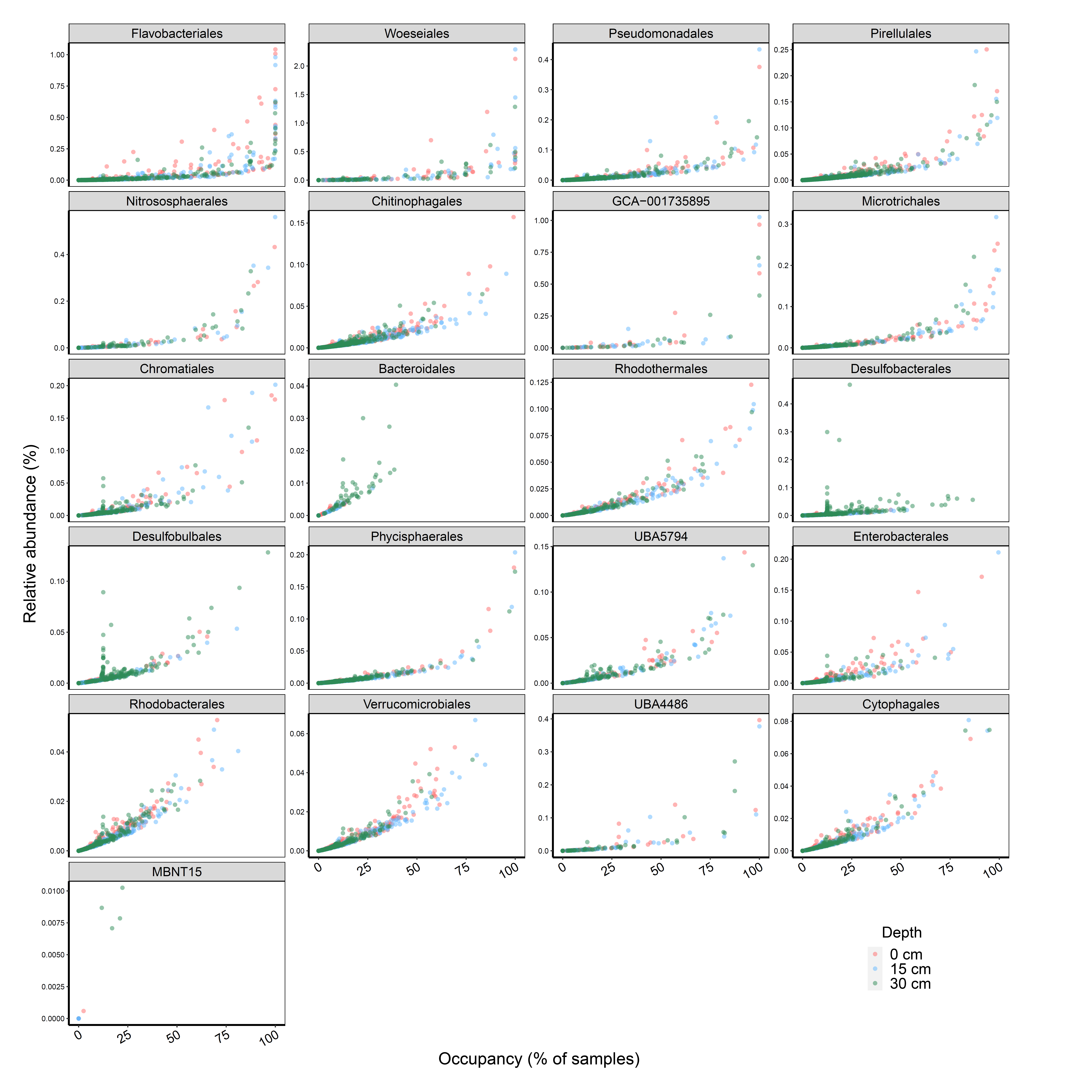


**Figure S5.** Relative abundance of the most abundant families **(a)** and genera **(b)** detected in the longitudinal survey. Results are shown for taxa that were taxonomically assigned based on the 16S rRNA gene amplicon data. Error bars show standard deviations of the mean and significance was tested using linear regression analyses with depth treated as a continuous variable (* *p* < 0.05, ** *p* < 0.01, *** *p* < 0.001). Also shown is the specialization index (SI) for each taxon based on the coefficient of variance of their relative abundance across the longitudinal 16S rRNA gene amplicon sequencing datasets. SIs below the community-wide SI means of 0.65 (family level) and 0.66 (genus level) indicate habitat generalists, SIs above these means indicate habitat specialists.

**
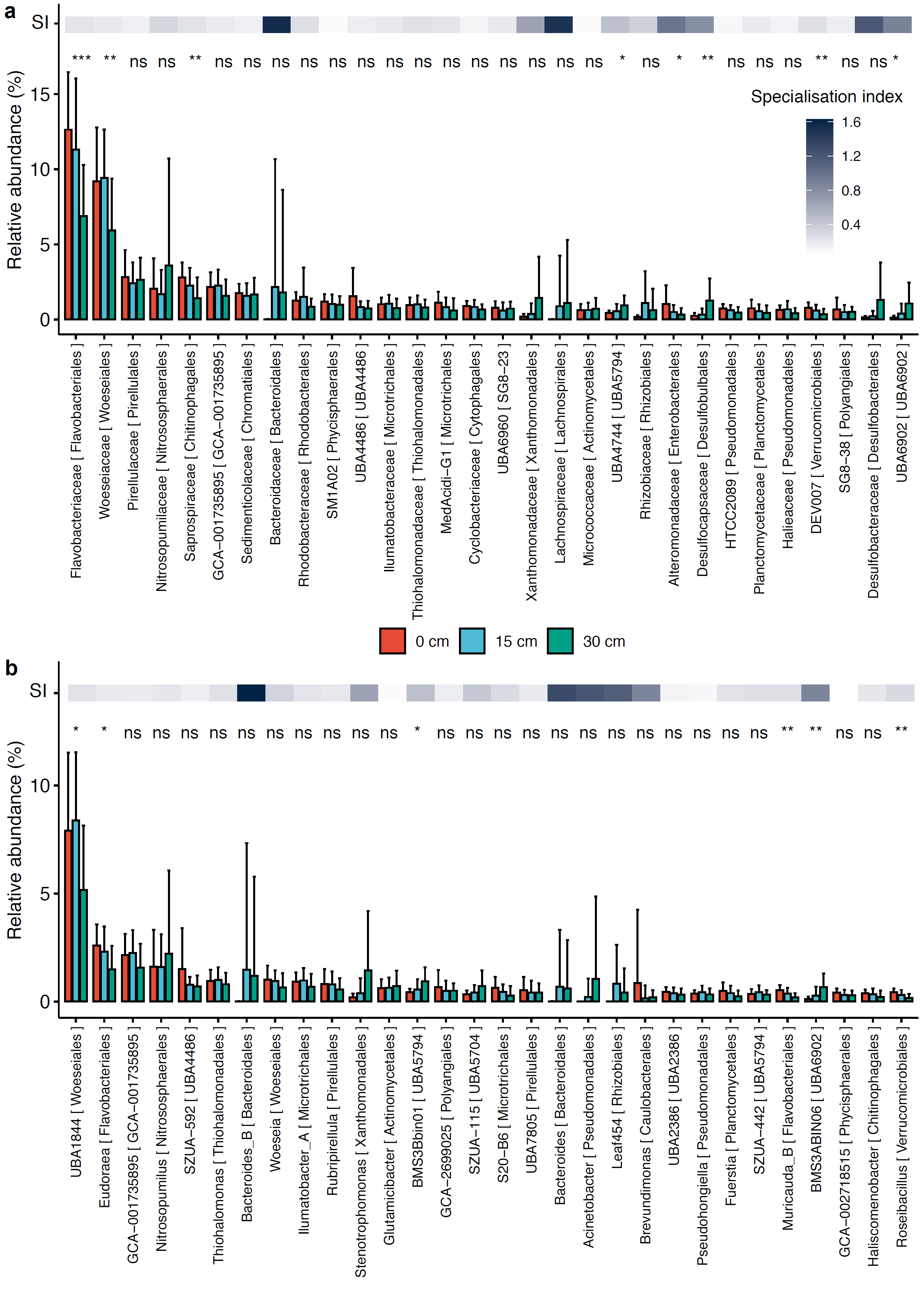
**

**Figure S6.** Occupancy of the amplicon sequence variants (ASVs) detected for each order across the dataset. Results are shown for the 20 most abundant orders and MBNT15. Each dot shows the percentage of samples in which each ASV was detected. Boxplots show medians, upper and lower quartiles, and the highest and lowest values that are within 1.5 times of the inter-quartile range.


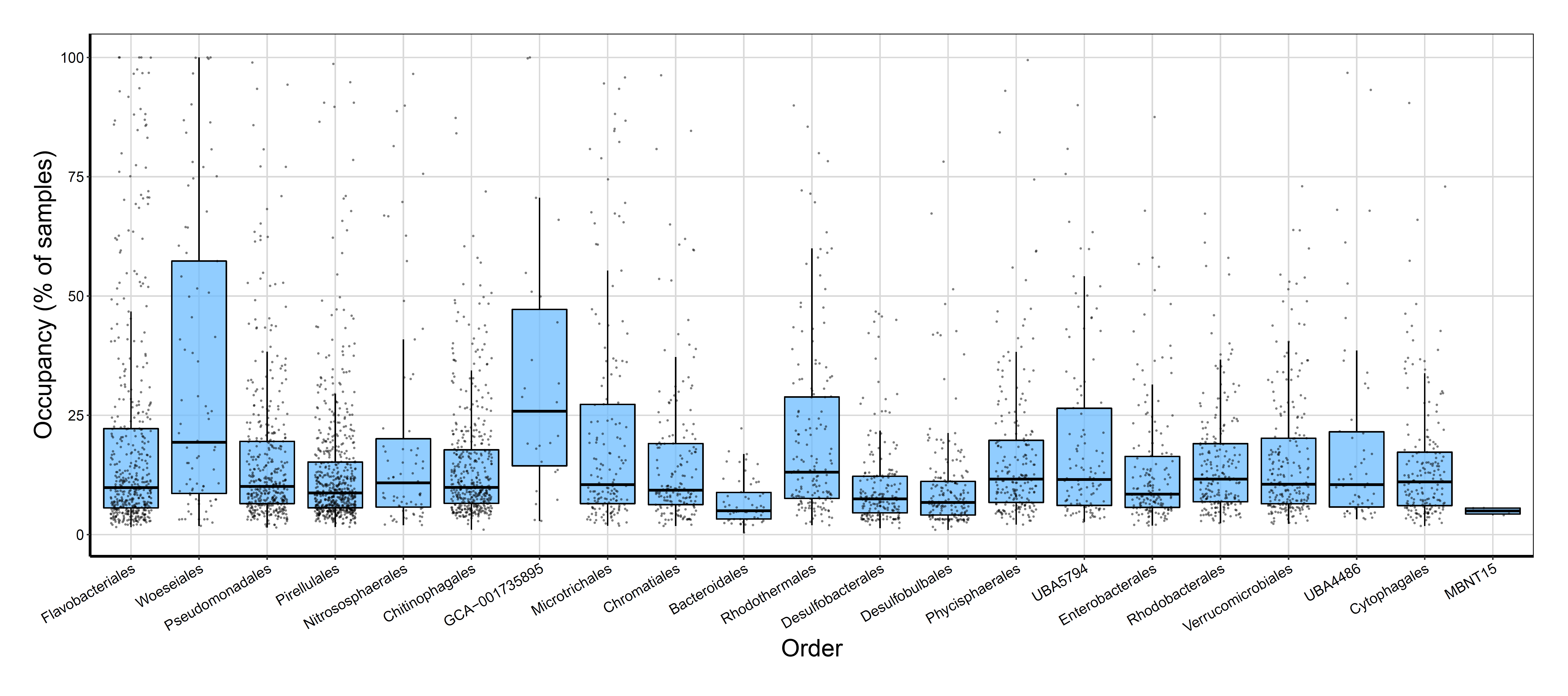


**Figure S7.** Comparison of phylum-level community composition across three different methods. **(a)** Community composition of permeable sediments collected across eight sampling times (A to H) based on 16S rRNA gene amplicon sequencing using the V4 region and primers F515/R806. **(b)** The first bar shows the phylum-level assignment of metagenome-assembled genomes (MAGs) based on GTDB-tk. The other bars show the community composition of permeable sediments collected at two sampling times (A and C) based on shotgun metagenomic sequencing and taxonomic assignment using the single-copy ribosomal marker gene *rplP*.


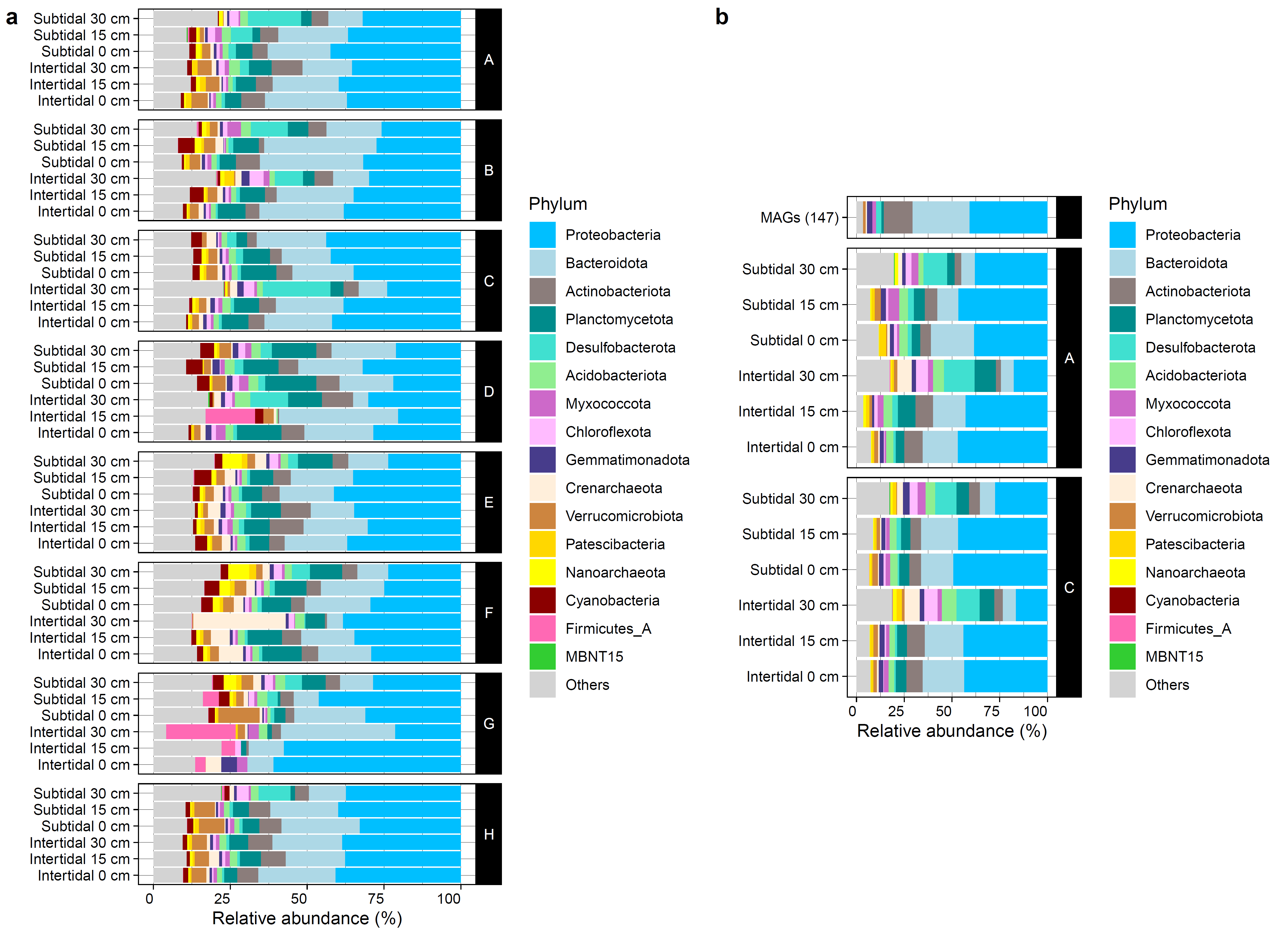


**Figure S8.** Maximum-likelihood tree of amino acid sequences of the sulfide-quinone oxidoreductase (Sqr), a marker for sulfide oxidation. The tree shows sequences from permeable sediment metagenome-assembled genomes (blue) alongside representative reference sequences (black). The subgroup of each reference sequence is denoted. The tree was constructed using the JTT matrix-based model, used all sites, and was bootstrapped with 50 replicates and midpoint-rooted.

**
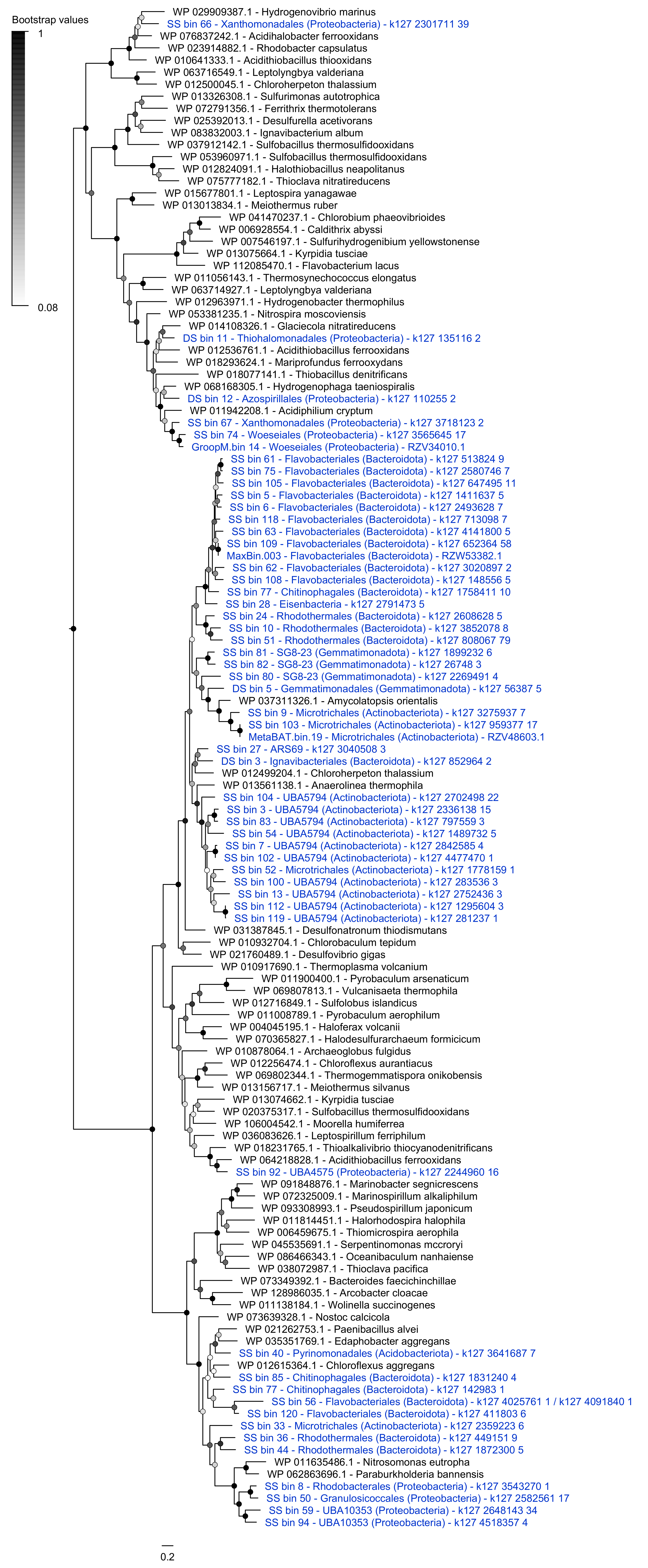
**

**Figure S9.** Maximum-likelihood tree of amino acid sequences of flavocytochrome *c* sulfide dehydrogenase (FCC), a marker for sulfide oxidation. The tree shows sequences from permeable sediment metagenome-assembled genomes (blue) alongside representative reference sequences (black). The tree was constructed using the JTT matrix-based model, used all sites, and was bootstrapped with 50 replicates and midpoint-rooted.

**
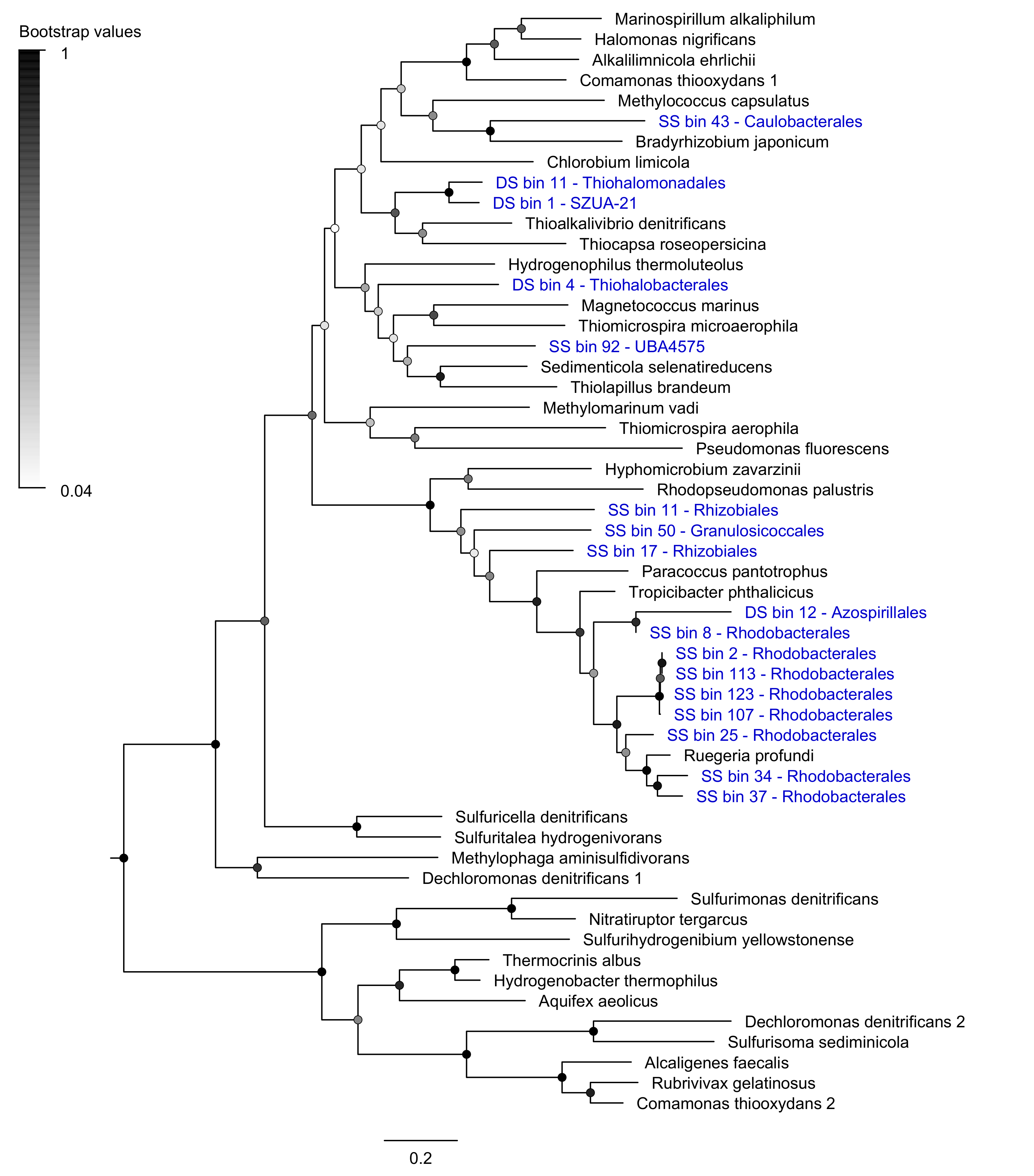
**

**Figure S10.** Maximum-likelihood tree of amino acid sequences of dissimilatory sulfite reductase A subunit (DsrA). The tree shows sequences from permeable sediment metagenome-assembled genomes (blue) alongside representative reference sequences (black). This enzyme is a marker for dissimilatory sulfite reduction (middle and bottom major clades; Desulfobacterota bins) and sulfide oxidation (top clade, rDsrA; Proteobacteria bins). The tree was constructed using the JTT matrix-based model, used all sites, and was bootstrapped with 50 replicates and midpoint-rooted.

**
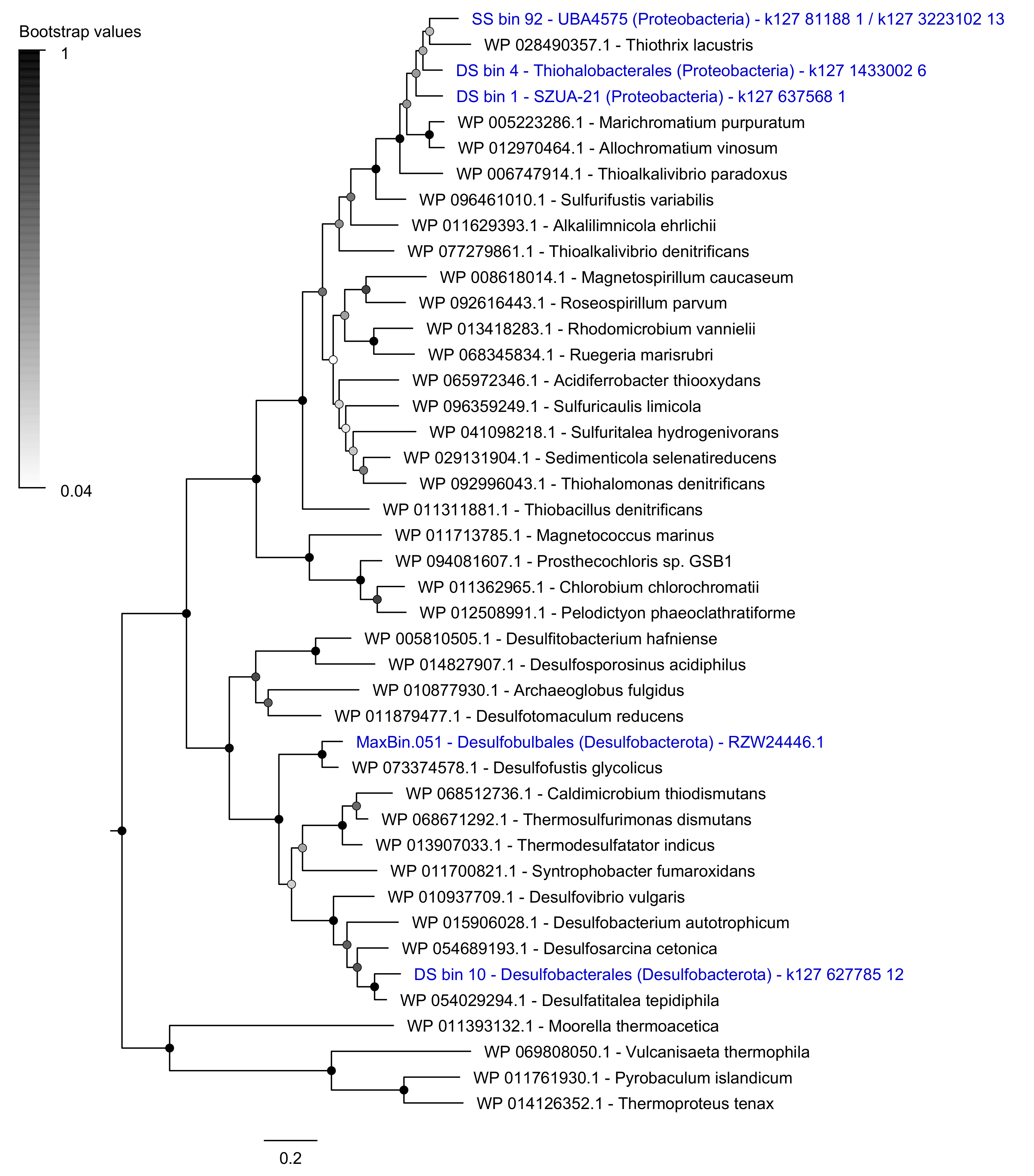
**

**Figure S11.** Maximum-likelihood tree of amino acid sequences of the thiosulfohydrolase (SoxB), a marker for thiosulfate oxidation. The tree shows sequences from permeable sediment metagenome-assembled genomes (blue) alongside representative reference sequences (black). The subgroup of each reference sequence is denoted. The tree was constructed using the JTT matrix-based model, used all sites, and was bootstrapped with 50 replicates and midpoint-rooted.

**
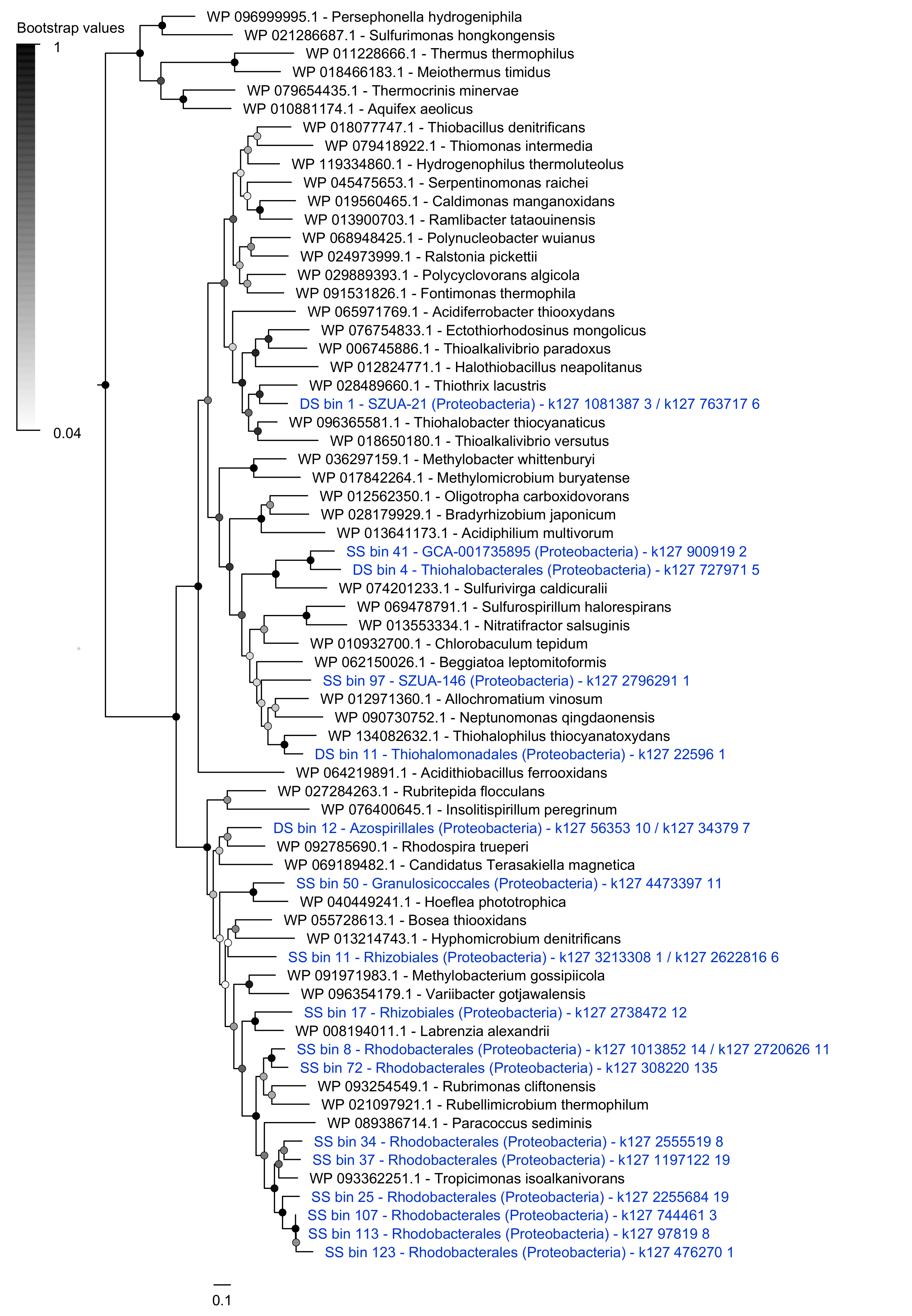
**

**Figure S12.** Maximum-likelihood tree of amino acid sequences of form I carbon monoxide dehydrogenase large subunit (CoxL), a marker for aerobic carbon monoxide oxidation. The tree shows sequences from permeable sediment metagenome-assembled genomes (blue) alongside representative reference sequences (black). The tree was constructed using the JTT matrix-based model, used all sites, and was bootstrapped with 50 replicates and midpoint-rooted.


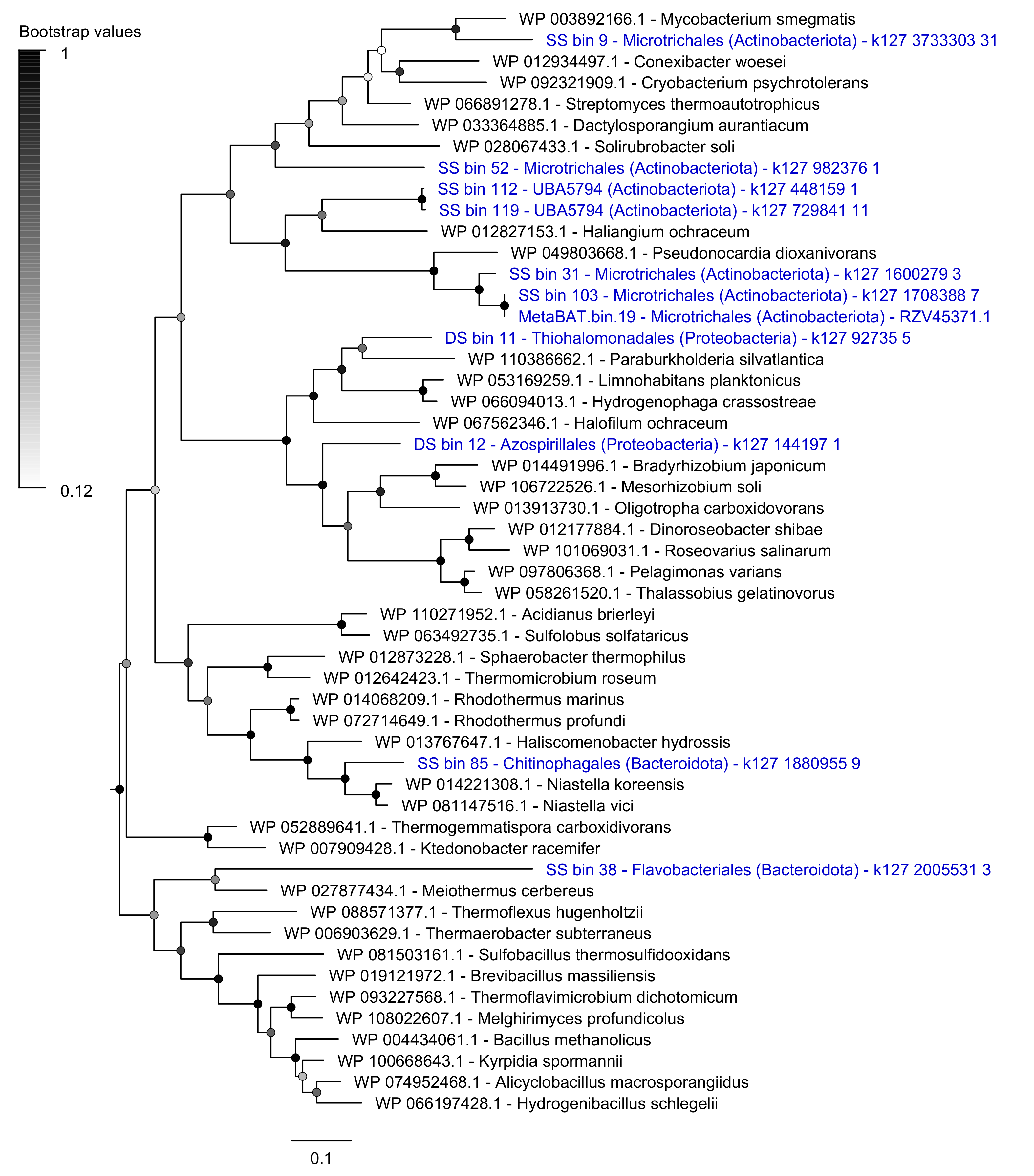


**Figure S13.** Maximum-likelihood tree of amino acid sequences of group 1 [NiFe]-hydrogenase large subunits, a marker for hydrogen oxidation during respiratory processes. The tree shows sequences from permeable sediment metagenome-assembled genomes (blue) alongside representative reference sequences (black). The subgroup of each reference sequence is denoted according to the HydDB classification scheme. The tree was constructed using the JTT matrix-based model, used all sites, and was bootstrapped with 50 replicates and midpoint-rooted.


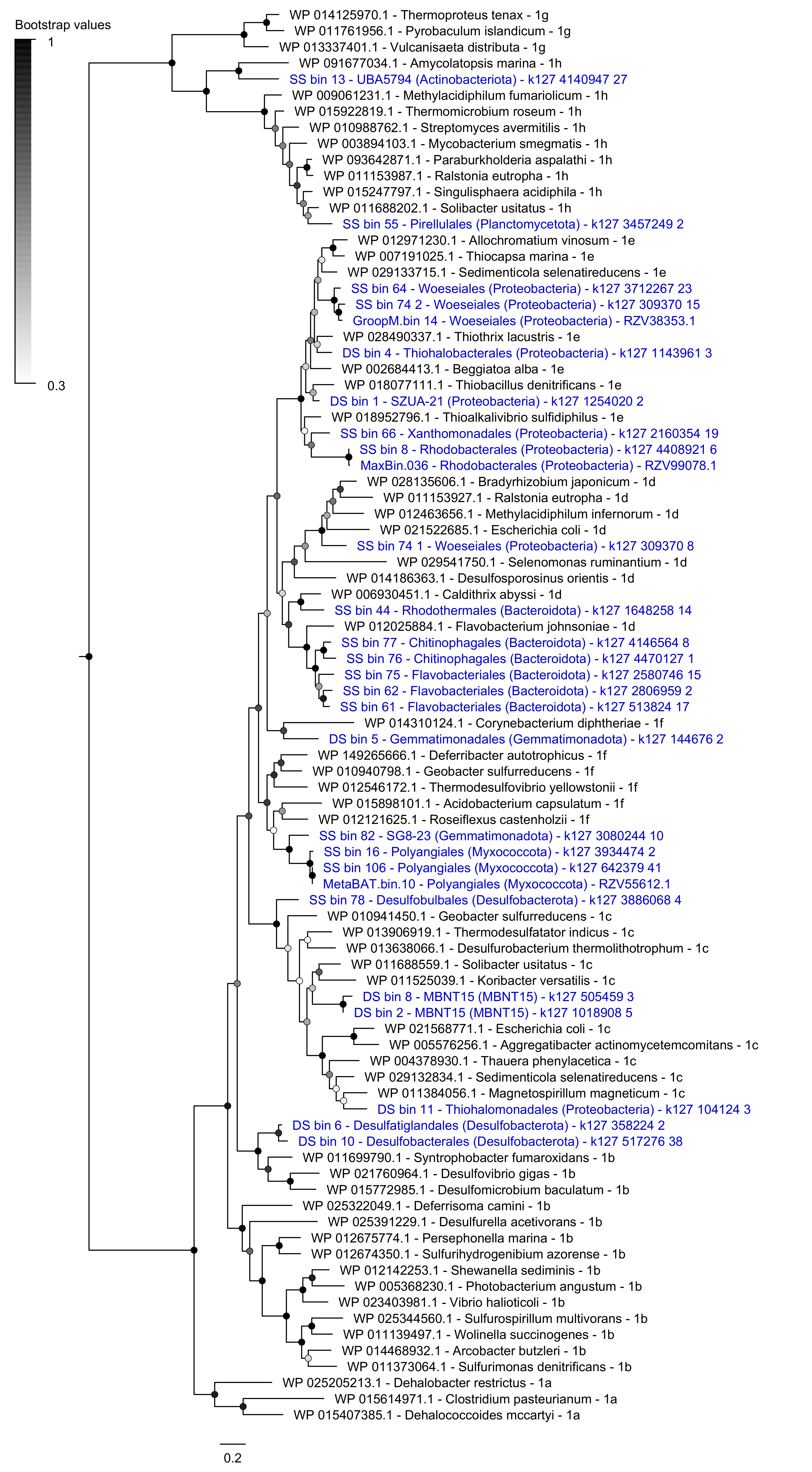


**Figure S14.** Maximum-likelihood tree of amino acid sequences of dissimilatory nitrate reductase G subunit (NarG), a marker for denitrification and dissimilatory nitrate reduction to ammonium. The tree shows sequences from permeable sediment metagenome-assembled genomes (blue) alongside representative reference sequences (black). The tree was constructed using the JTT matrix-based model, used all sites, and was bootstrapped with 50 replicates and midpoint-rooted.

**
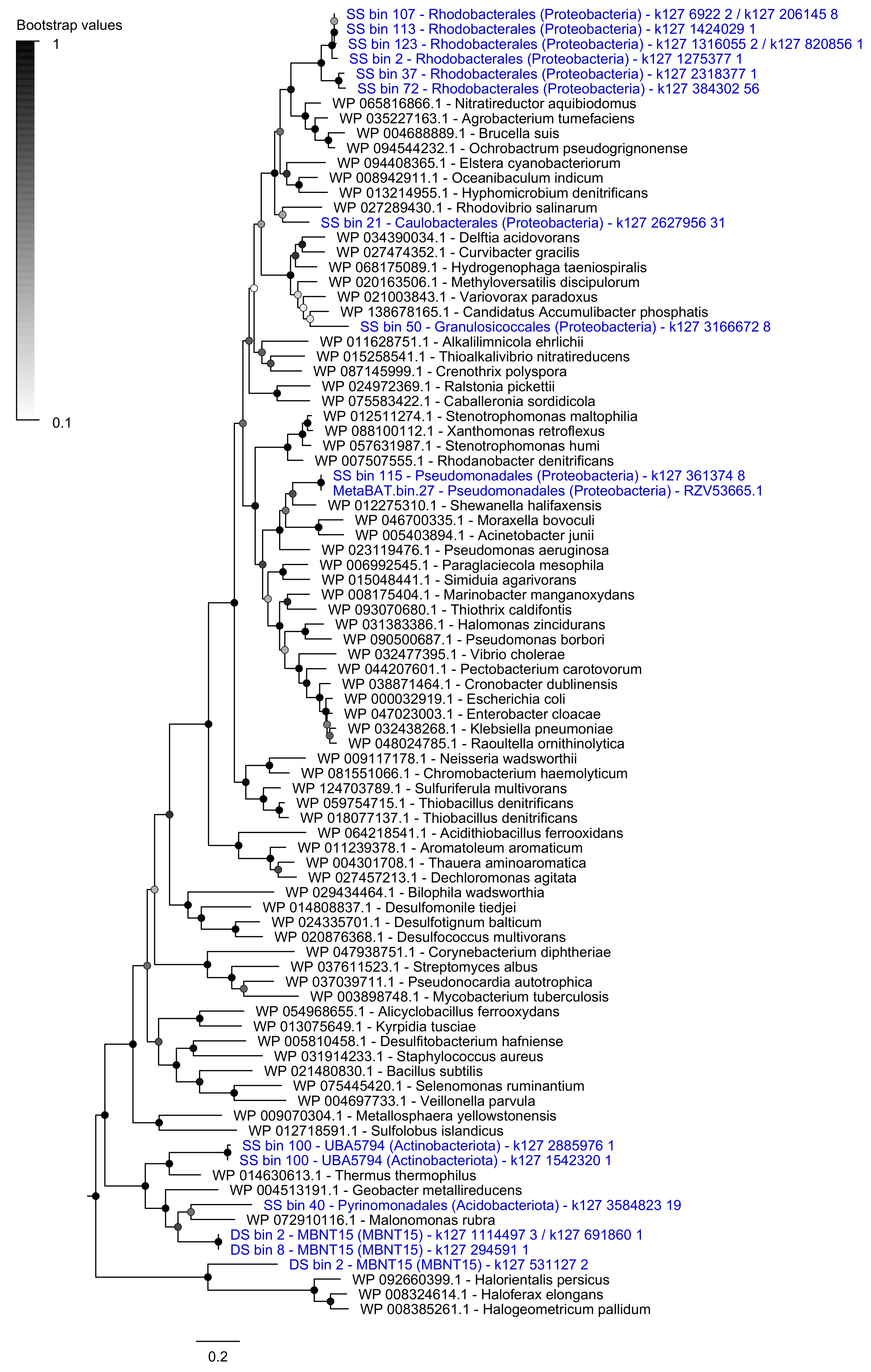
**

**Figure S15.** Maximum-likelihood tree of amino acid sequences of periplasmic nitrate reductase A subunit (NapA), a marker for denitrification and dissimilatory nitrate reduction to ammonium. The tree shows sequences from permeable sediment metagenome-assembled genomes (blue) alongside representative reference sequences (black). The tree was constructed using the JTT matrix-based model, used all sites, and was bootstrapped with 50 replicates and midpoint-rooted.


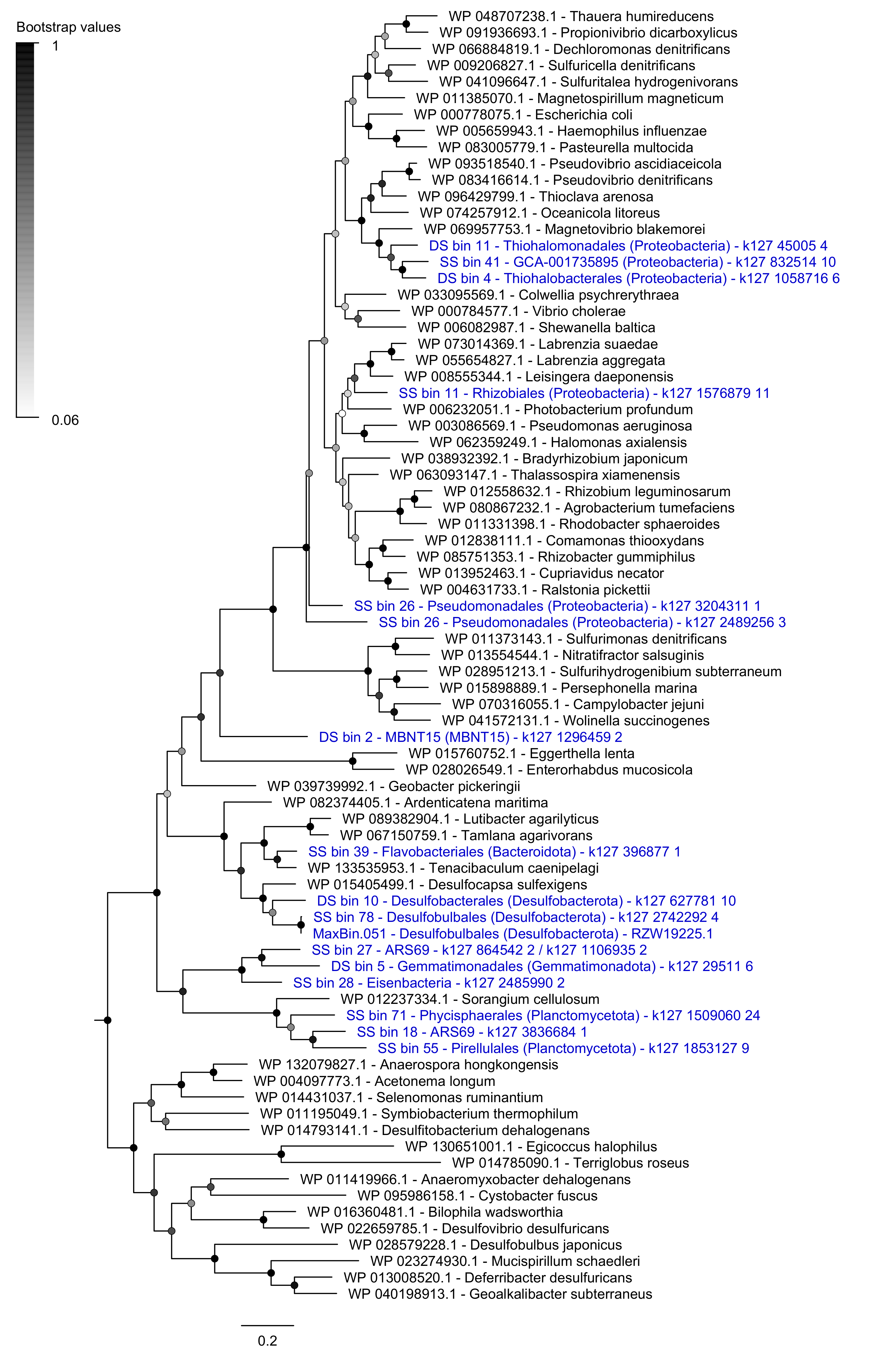


**Figure S16.** Maximum-likelihood tree of amino acid sequences of copper-containing nitrite reductase K subunit (NirK), a marker for denitrification. The tree shows sequences from permeable sediment metagenome-assembled genomes (blue) alongside representative reference sequences (black). The tree was constructed using the JTT matrix-based model, used all sites, and was bootstrapped with 50 replicates and midpoint-rooted.

**
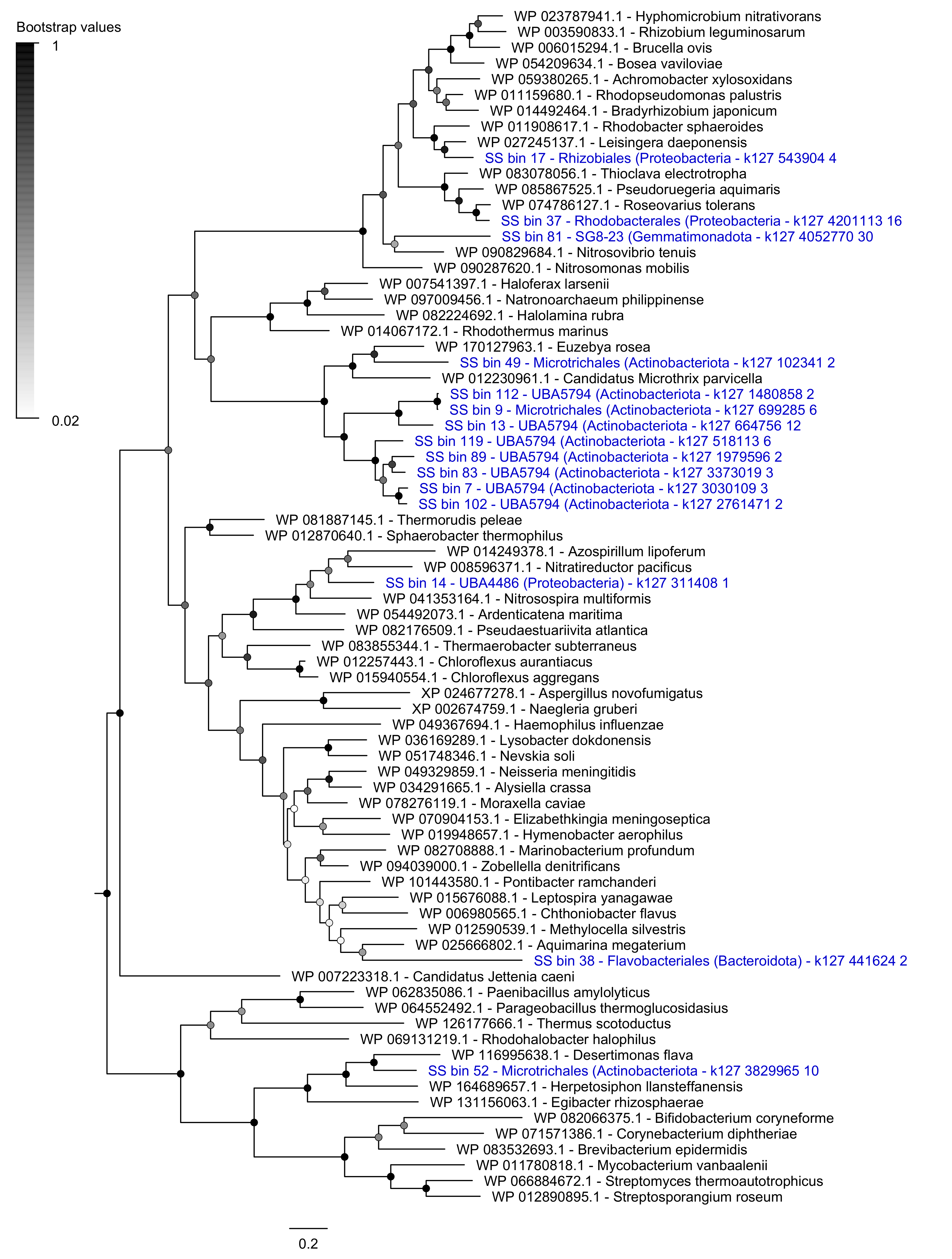
**

**Figure S17.** Maximum-likelihood tree of amino acid sequences of cytochrome *cd*_1_ nitrite reductase S subunit (NirS), a marker for denitrification. The tree shows sequences from permeable sediment metagenome-assembled genomes (blue) alongside representative reference sequences (black). The tree was constructed using the JTT matrix-based model, used all sites, and was bootstrapped with 50 replicates and midpoint-rooted.

**
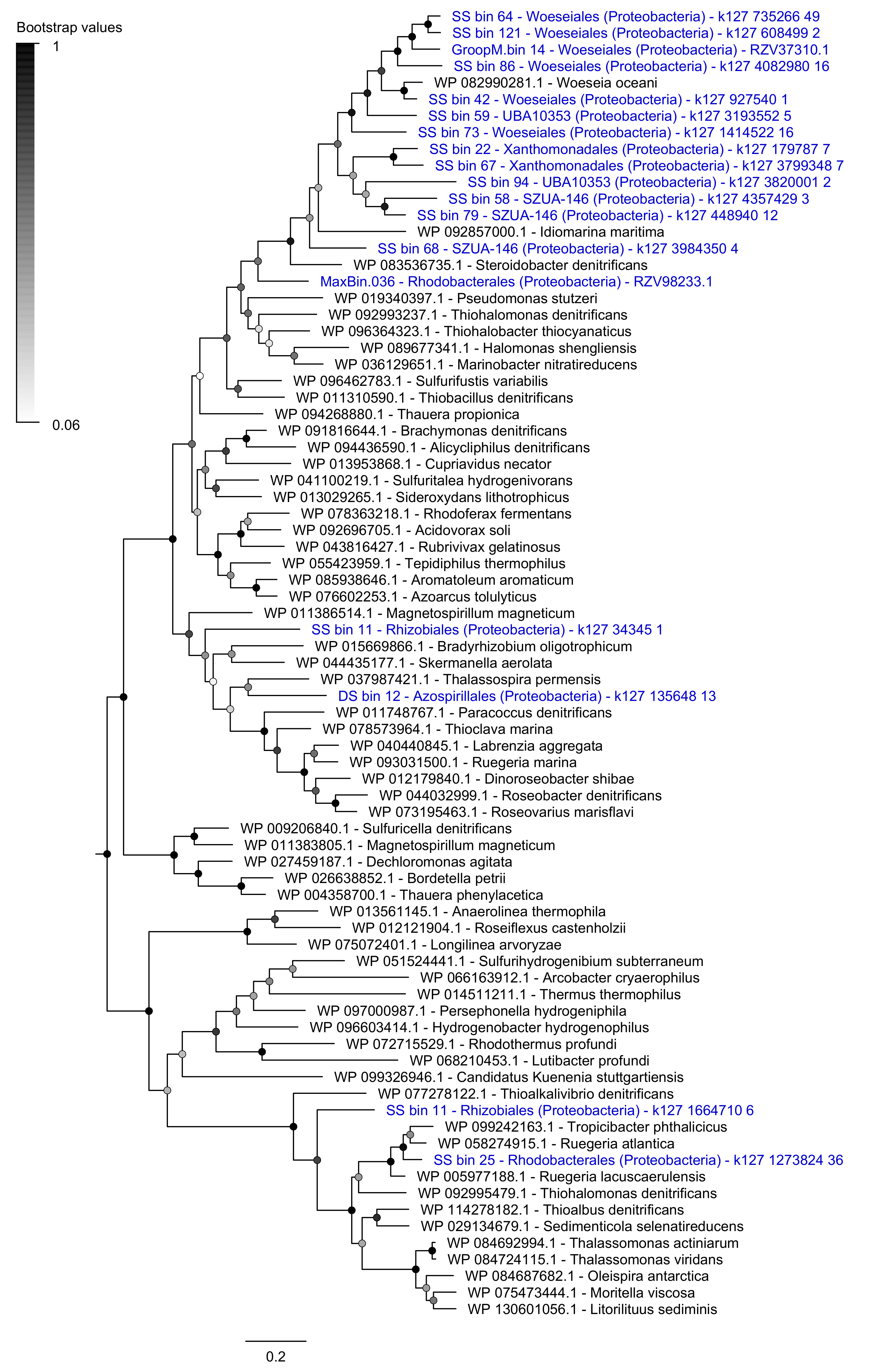
**

**Figure S18.** Maximum-likelihood tree of amino acid sequences of nitric oxide reductase B subunit (NorB), a marker for nitric oxide reduction. The tree shows sequences from permeable sediment metagenome-assembled genomes (blue) alongside representative reference sequences (black). The tree was constructed using the JTT matrix-based model, used all sites, and was bootstrapped with 50 replicates and midpoint-rooted.

**
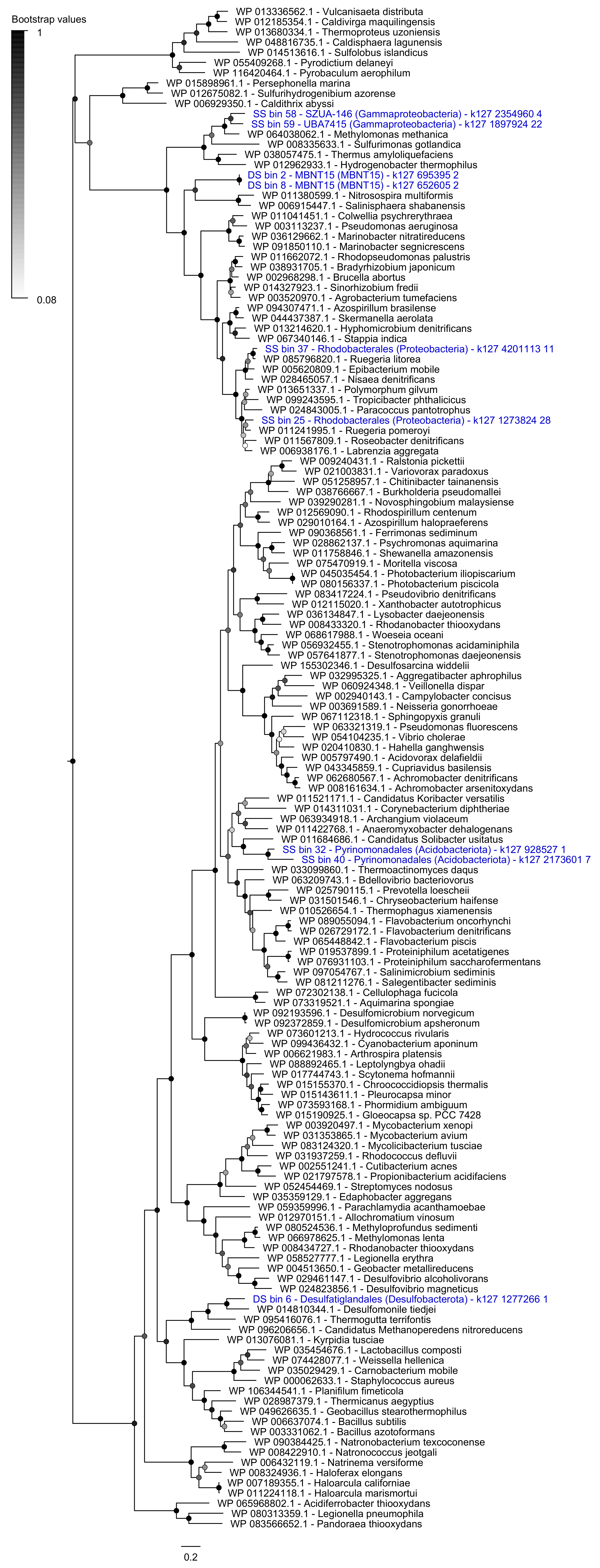
**

**Figure S19.** Maximum-likelihood tree of amino acid sequences of nitrous oxide reductase Z subunit (NosZ), a marker for nitrous oxide reduction. The tree shows sequences from permeable sediment metagenome-assembled genomes (blue) alongside representative reference sequences (black). The tree was constructed using the JTT matrix-based model, used all sites, and was bootstrapped with 50 replicates and midpoint-rooted.

**
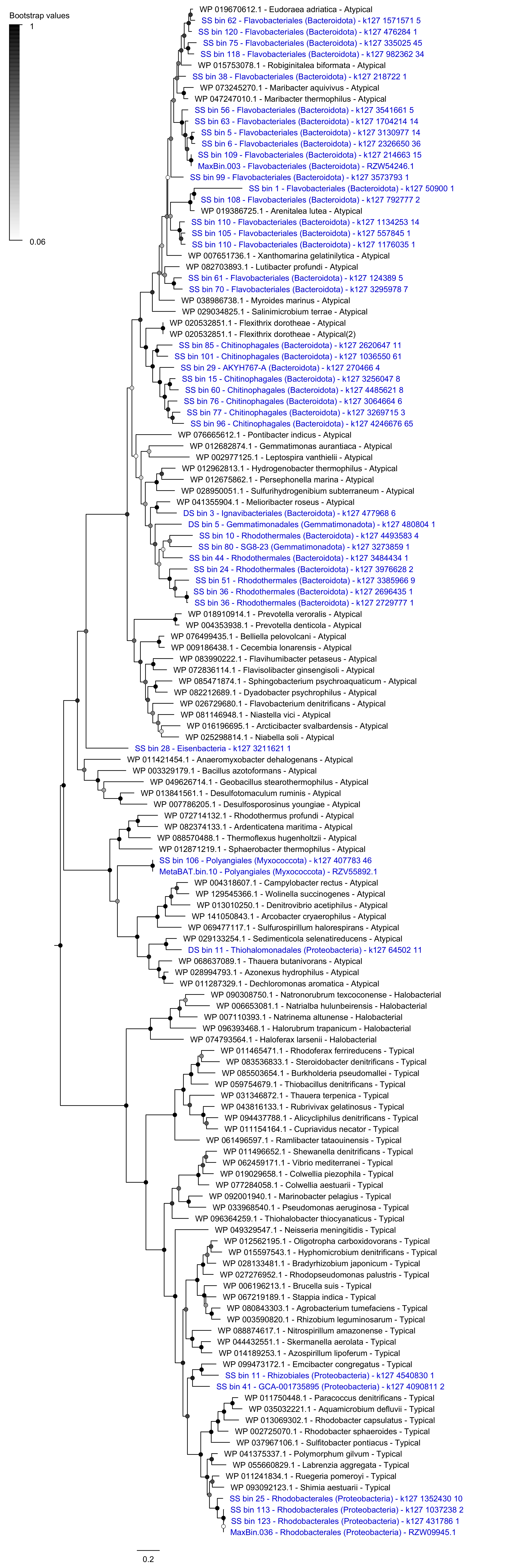
**

**Figure S20.** Maximum-likelihood tree of amino acid sequences of ammonifying nitrite reductase A subunit (NrfA), a marker for dissimilatory nitrate reduction to ammonium. The tree shows sequences from permeable sediment metagenome-assembled genomes (blue) alongside representative reference sequences (black). The tree was constructed using the JTT matrix-based model, used all sites, and was bootstrapped with 50 replicates and midpoint-rooted.

**
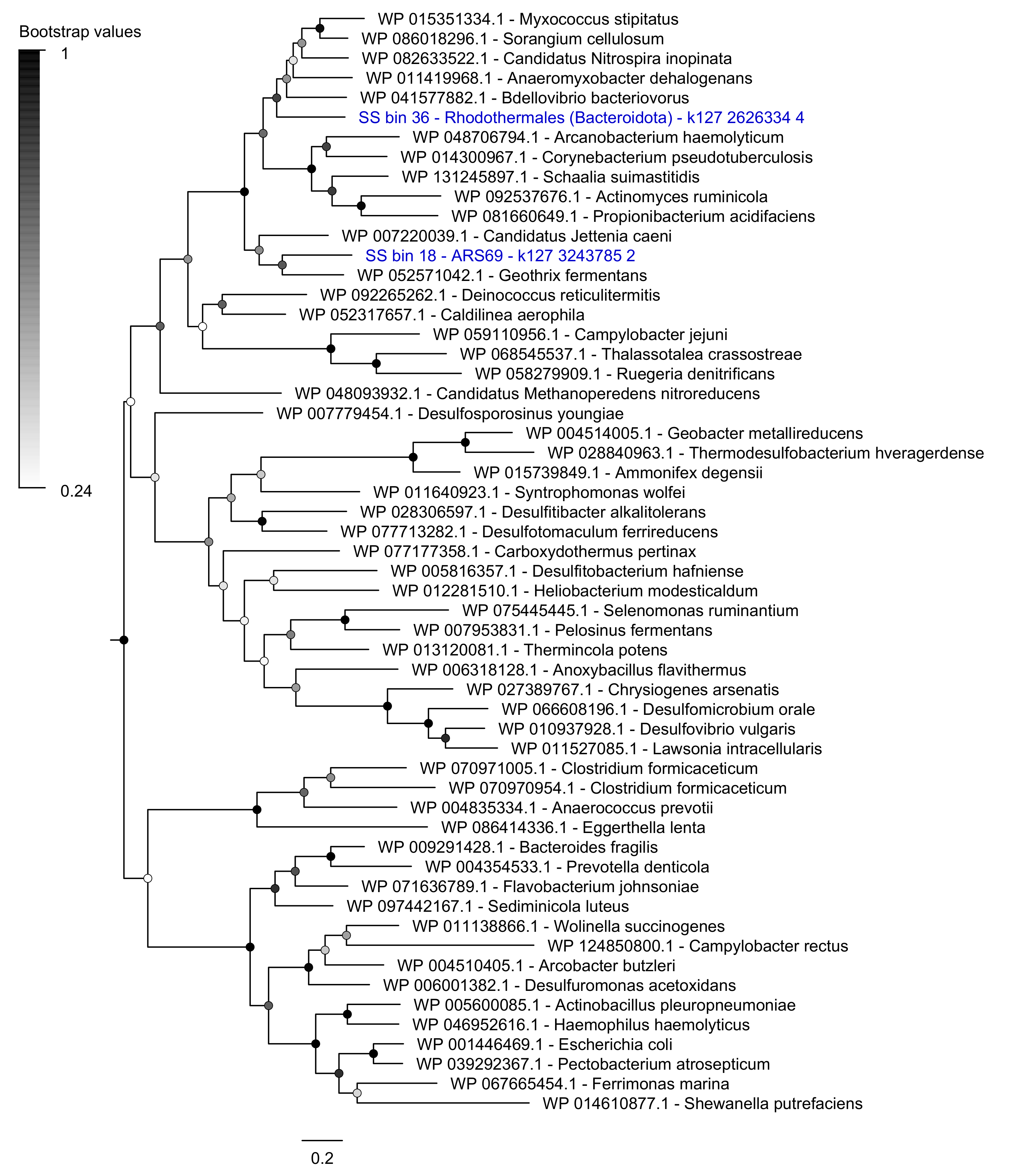
**

**Figure S21.** Maximum-likelihood tree of amino acid sequences of group 3 [NiFe]-hydrogenase large subunits, a marker for hydrogen production during fermentation processes. The tree shows sequences from permeable sediment metagenome-assembled genomes (blue) alongside representative reference sequences (black). The subgroup of each reference sequence is denoted according to the HydDB classification scheme. The tree was constructed using the JTT matrix-based model, used all sites, and was bootstrapped with 50 replicates and midpoint-rooted.

**
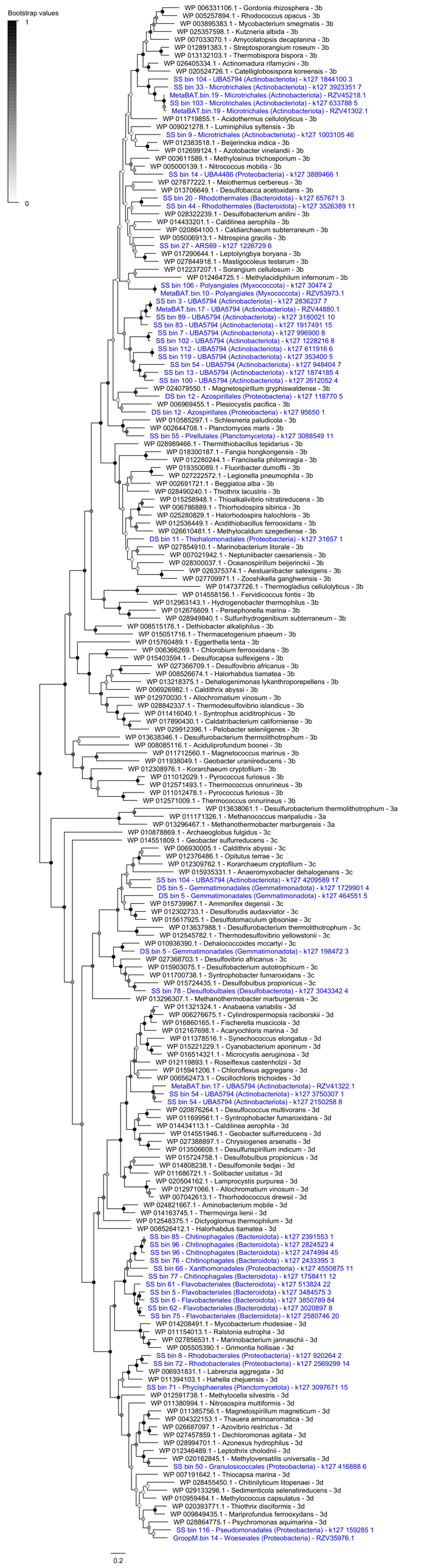
**

**Figure S22.** Maximum-likelihood tree of amino acid sequences of metal-reducing decaheme-associated outer membrane protein (MtrB), a marker for iron(III) reduction. The tree shows sequences from permeable sediment metagenome-assembled genomes (blue) alongside representative reference sequences (black). The tree was constructed using the JTT matrix-based model, used all sites, and was bootstrapped with 50 replicates and midpoint-rooted. Note no binned reads for OmcB, an iron-reducing outer membrane protein in *Geobacter* and *Desulfuromonas* species, were detected.


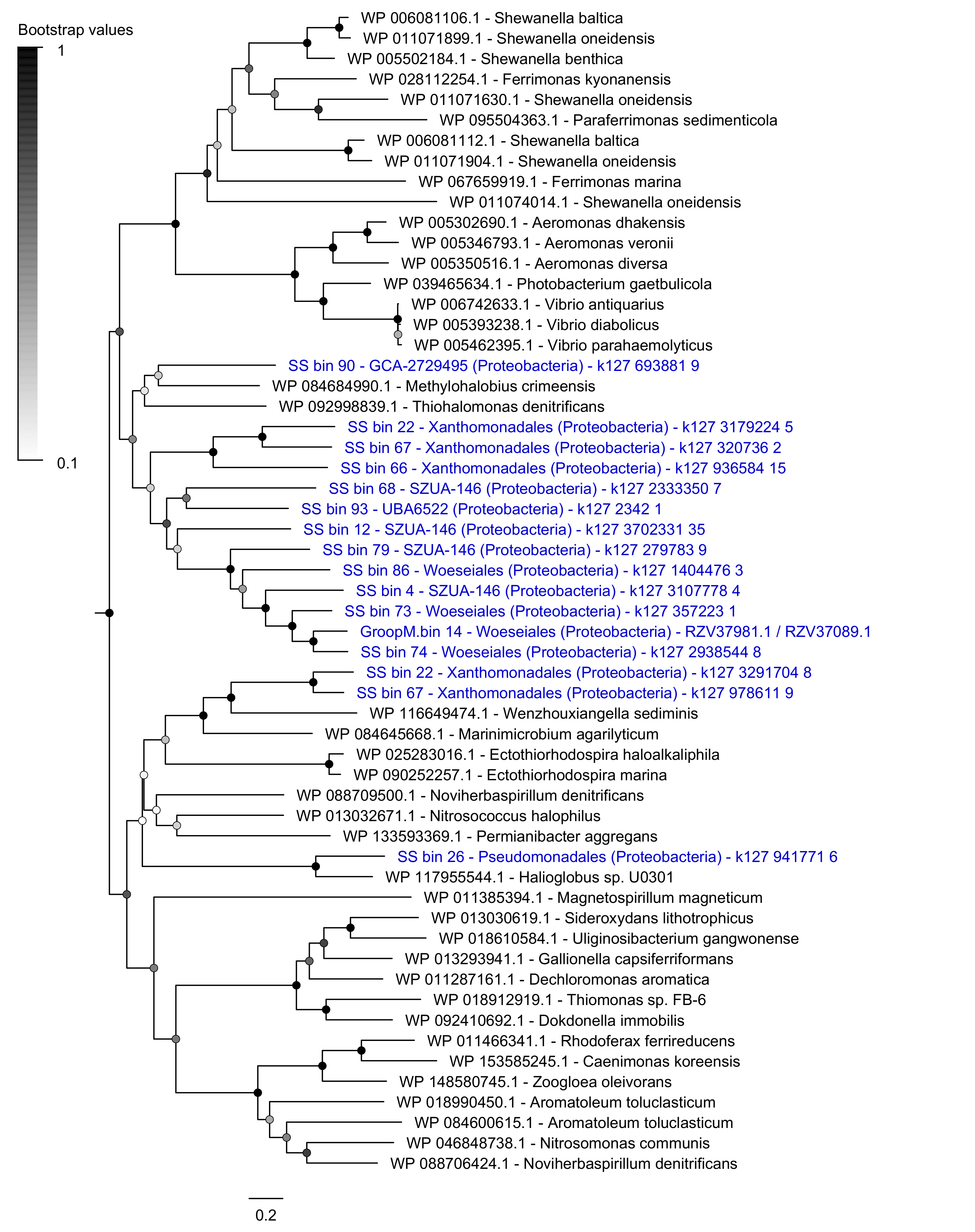


**Figure S23.** Maximum-likelihood tree of amino acid sequences of reductive dehalogenase A subunit (RdhA), a marker for the dehalogenation of diverse organohalide compounds. The tree shows sequences from permeable sediment metagenome-assembled genomes (blue) alongside representative reference sequences (black). The tree was constructed using the JTT matrix-based model, used all sites, and was bootstrapped with 50 replicates and midpoint-rooted.

**
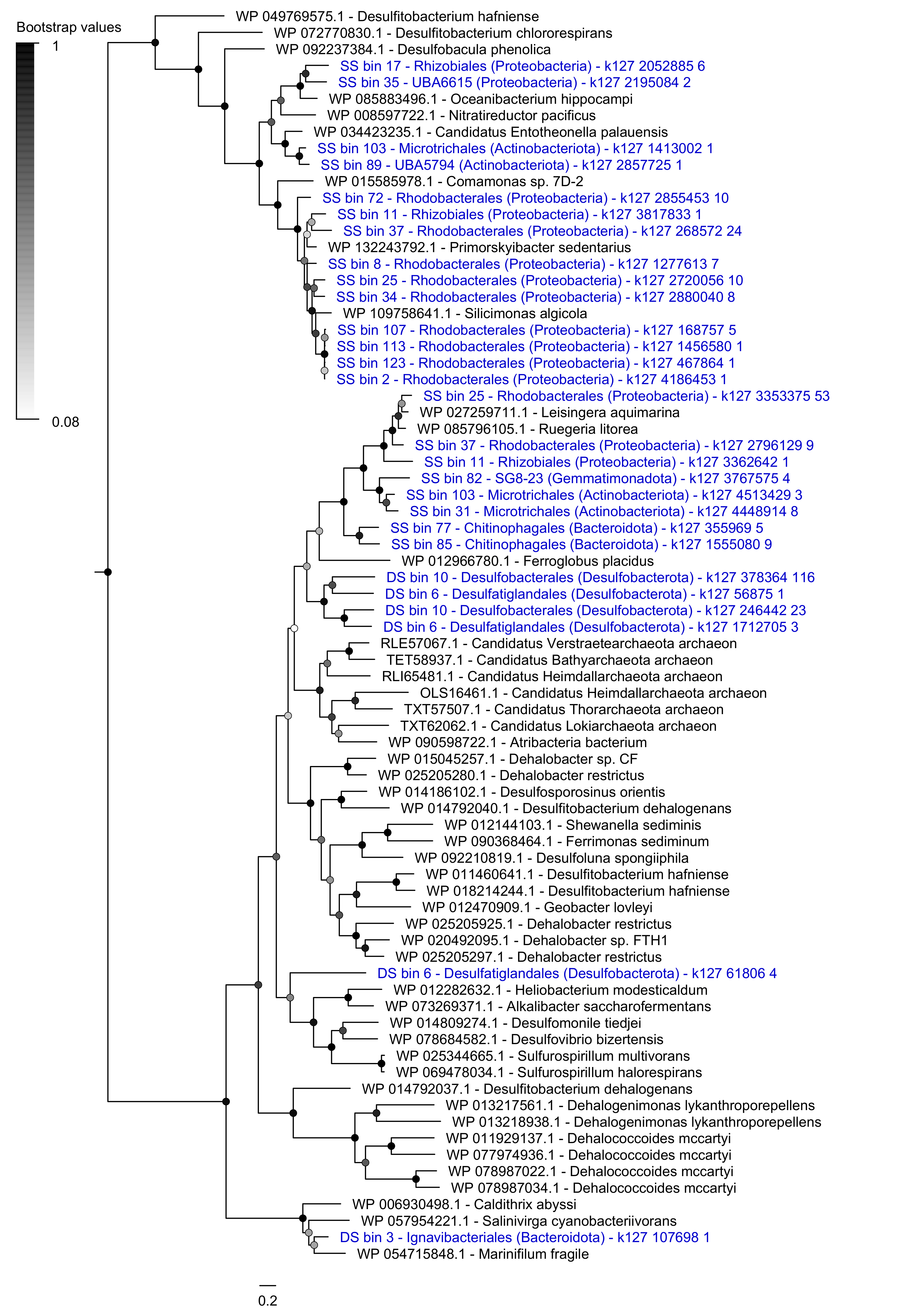
**

**Figure S24.** Maximum-likelihood tree of amino acid sequences of fumarate reductase A subunit (FrdA), a marker for fumarate reduction. The tree shows sequences from permeable sediment metagenome-assembled genomes (blue) alongside representative reference sequences (black). The tree was constructed using the JTT matrix-based model, used all sites, and was bootstrapped with 50 replicates and midpoint-rooted. Note only canonical fumarate reductases are shown and it is possible that homologous enzymes within the complex II superfamily also mediate fumarate reduction either reversibly or unidirectionally.


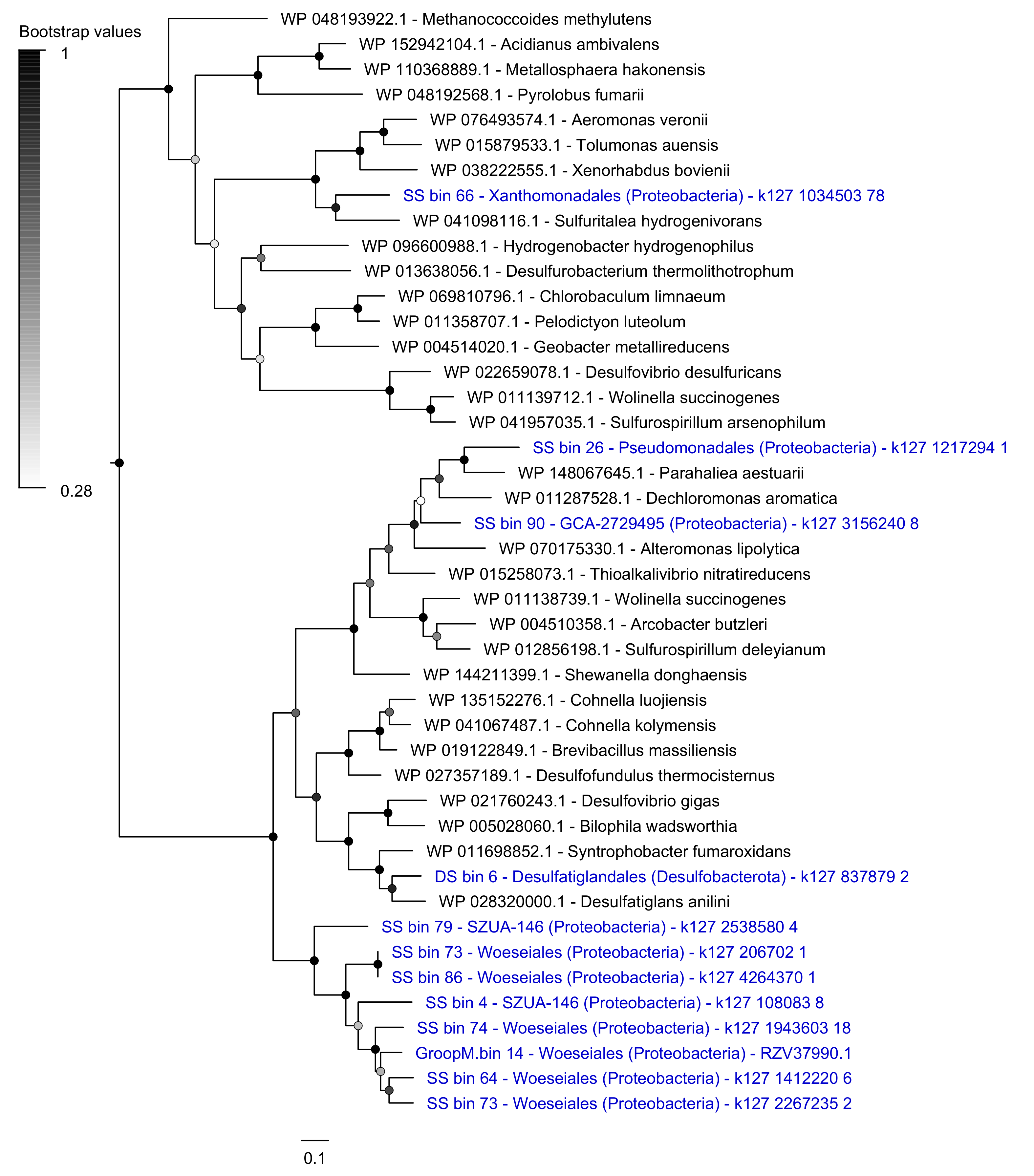


**Figure S25.** Maximum-likelihood tree of amino acid sequences of ribulose 1,5-bisphosphate carboxylase / oxygenase (RuBisCO) large subunit (RbcL), a marker for carbon fixation through the Calvin-Benson-Bassham cycle. The tree shows sequences from permeable sediment metagenome-assembled genomes (blue) alongside representative reference sequences (black). The tree was constructed using the JTT matrix-based model, used all sites, and was bootstrapped with 50 replicates and midpoint-rooted.

**
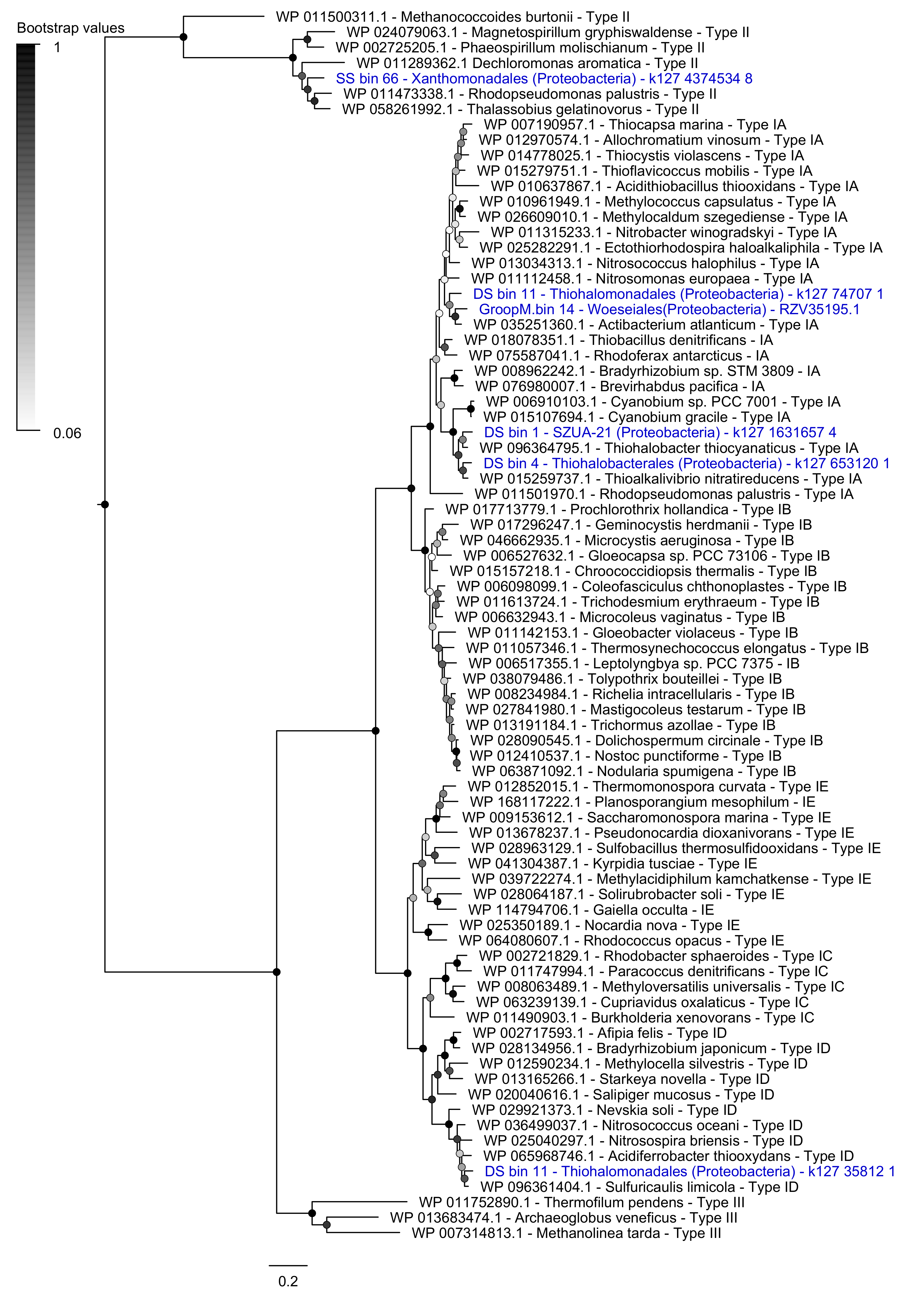
**

**Figure S26.** Maximum-likelihood tree of amino acid sequences of microbial rhodopsins. Only clades known to support energy transduction through coupling photon capture to proton or sodium translocation are shown. The tree shows sequences from permeable sediment metagenome-assembled genomes (blue) alongside representative reference sequences (black). The tree was constructed using the JTT matrix-based model, used all sites, and was bootstrapped with 50 replicates and midpoint-rooted.

**
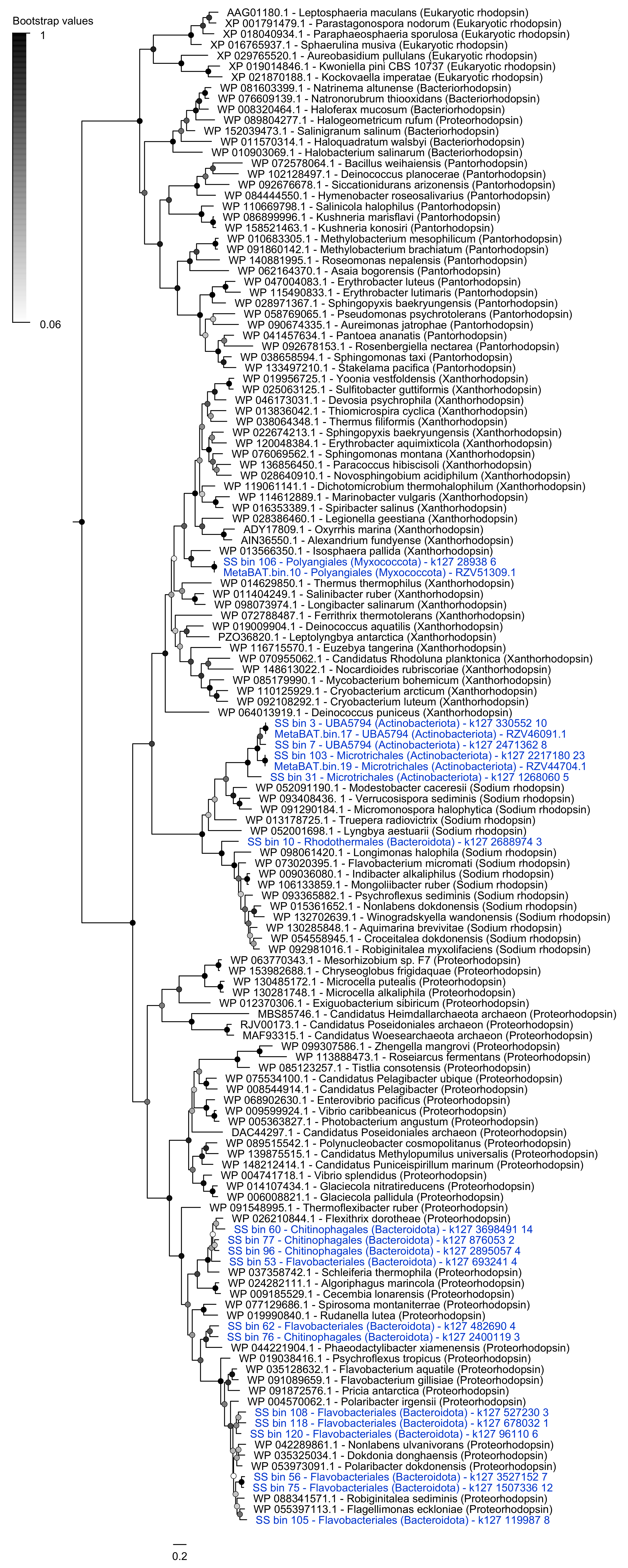
**

**Figure S27.** Maximum-likelihood tree of amino acid sequences of acetyl-CoA synthase B subunit (AcsB), a marker gene for acetate oxidation. The tree shows sequences from permeable sediment metagenome-assembled genomes (blue) alongside representative reference sequences (black). The tree was constructed using the JTT matrix-based model, used all sites, and was bootstrapped with 50 replicates and midpoint-rooted. Note that this enzyme is a marker for both homoacetogenesis (reductive Wood-Ljungdahl pathway) and acetate oxidation (oxidative Wood-Ljungdahl pathway), but generally acts in the oxidative direction in sulfate-reducing bacteria.

**
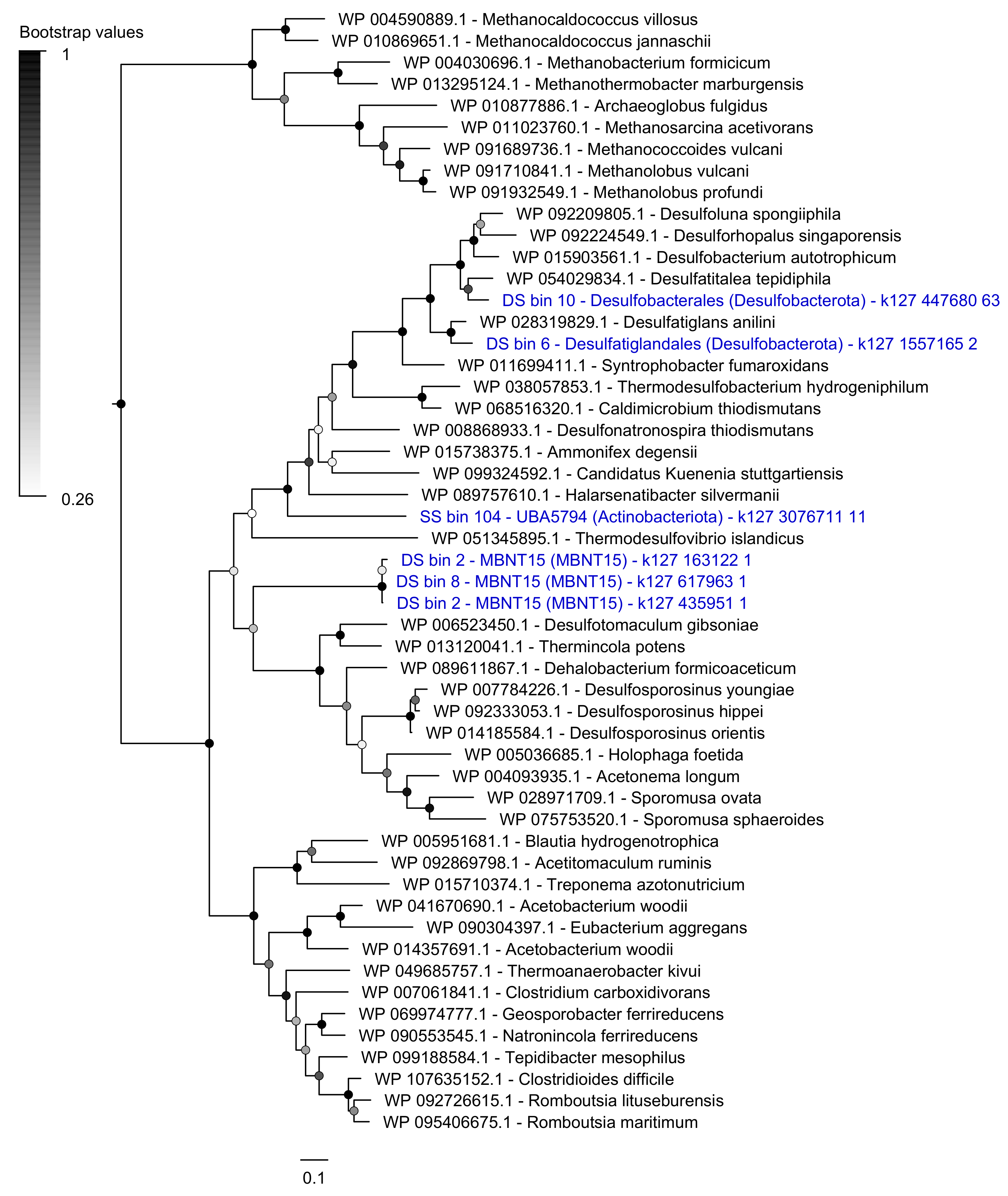
**

**Figure S28.** Relative abundance of the most abundant families and genera detected in the microcosm experiments. The relative abundance of the most abundant taxonomically assigned families **(a)** and genera **(b)** from surface (top) and deep (bottom) sands is depicted with red bars. The changes of their relative abundance is shown after sampled sands (red bars) were incubated in slurries for two weeks in one of three conditions: continual light oxic conditions (light blue bars), continual dark anoxic conditions (green bars), or disrupted conditions (dark blue bars) in which slurries were shifted between light oxic and dark anoxic conditions every 24 hours. Error bars show standard deviations of the mean and significance was tested using one-way ANOVAs (* *p* < 0.05, ** *p* < 0.01, *** *p* < 0.001, **** *p* < 0.0001, ns *p* > 0.05 (not significant)). The heatmap depicts the specialization index (SI) for each taxon based on the coefficient of variance of their relative abundance across the longitudinal study depicted in **Fig. 1**; SIs below the community-wide SI means of 0.65 (family level) and 0.66 (genus level) indicate habitat generalists, SIs above these means indicate habitat specialists.


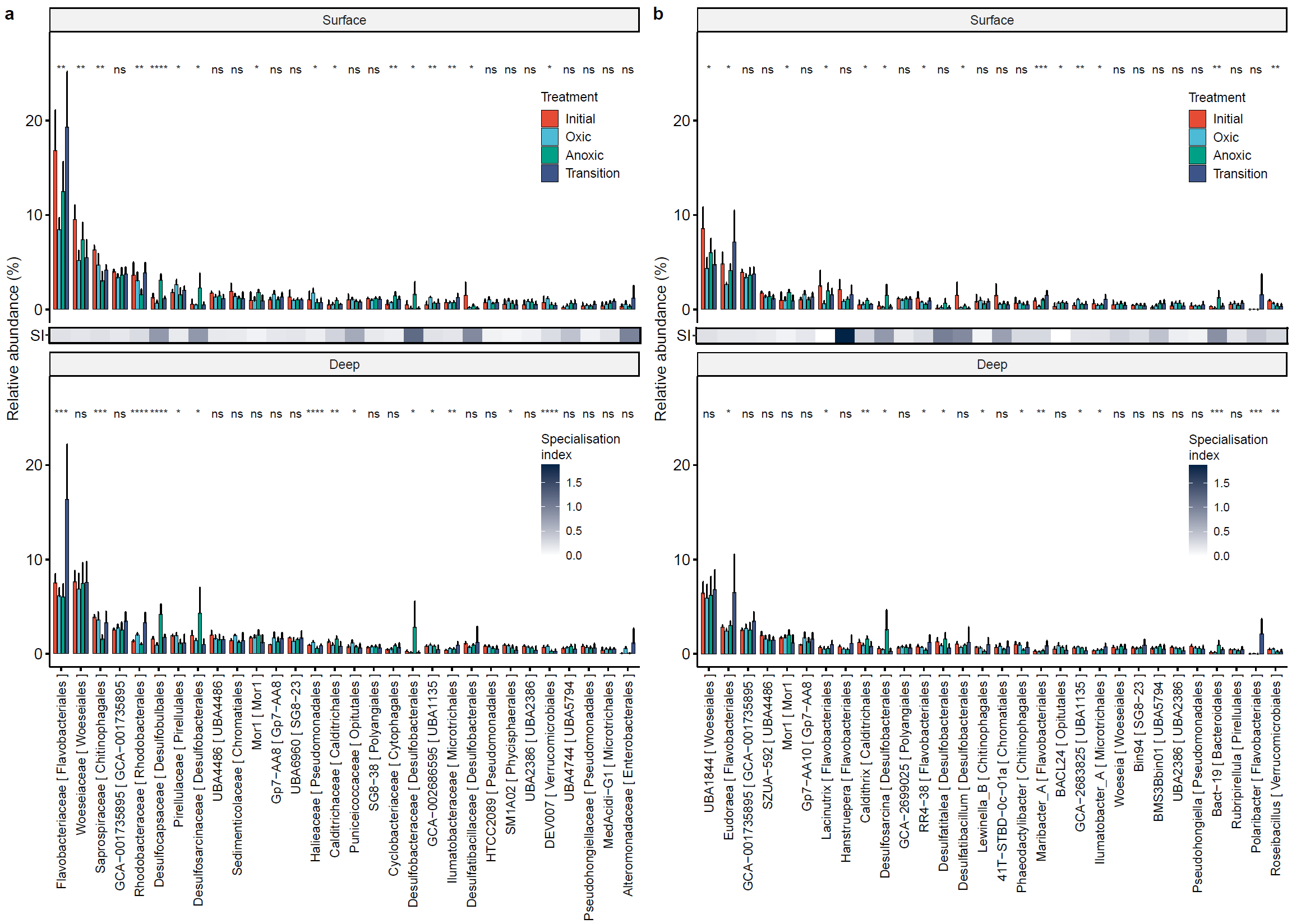


**Figure S29.** Maximum-likelihood tree of amino acid sequences of photosystem II subunit A (PsbA), a marker gene for photophosphorylation. The tree shows sequences from permeable sediment metagenome-assembled genomes (blue) and unbinned contigs likely originating from Rhodobacteraceae and diatom chloroplasts (red) alongside representative reference sequences (black). The tree was constructed using the JTT matrix-based model, used all sites, and was bootstrapped with 50 replicates and midpoint-rooted.


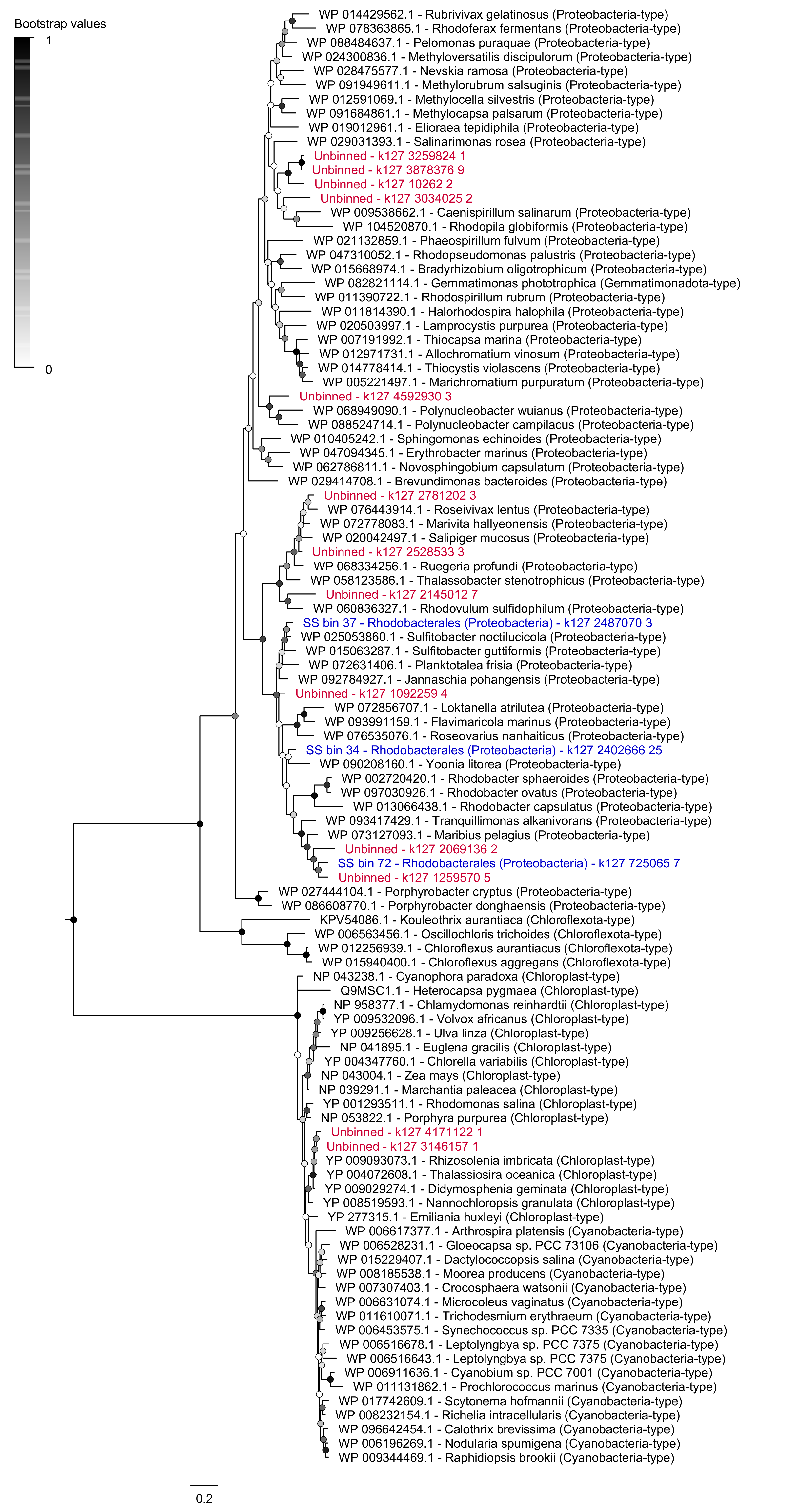

Supplement: Supplementary file 1 — Supplementary information [file 41396_2021_988_MOESM1_ESM.docx]
